# Microneedles as an Emerging Platform for Transdermal Delivery of Phytochemicals

**DOI:** 10.1021/acs.molpharmaceut.4c00894

**Published:** 2024-10-29

**Authors:** B.H. Jaswanth Gowda, Mohammed Gulzar Ahmed, Raghu Raj Singh Thakur, Ryan F. Donnelly, Lalitkumar K. Vora

**Affiliations:** † School of Pharmacy, Queen’s University Belfast, Medical Biology Centre, Belfast BT9 7BL, United Kingdom; ‡ Department of Pharmaceutics, Yenepoya Pharmacy College & Research Centre, Yenepoya (Deemed to be University), Mangalore 575018, Karnataka, India

**Keywords:** Microneedles, Herbal drugs, Phytoconstituents, Phytochemicals, Intradermal, Transdermal

## Abstract

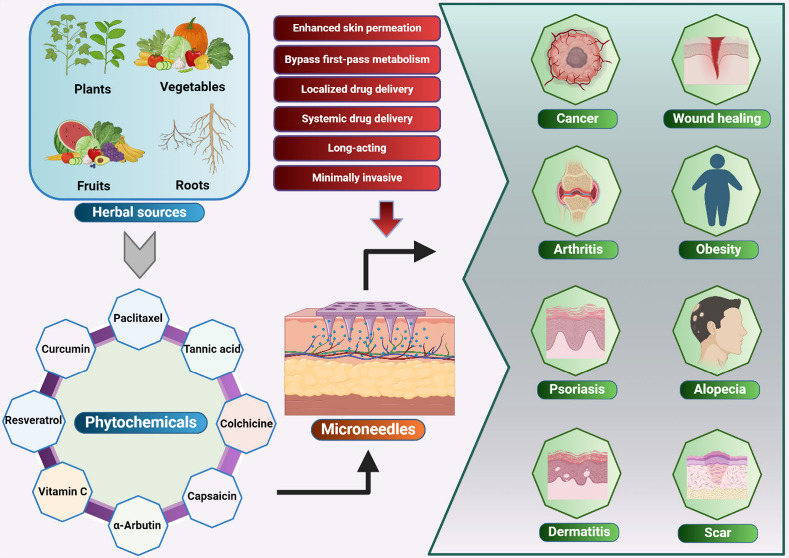

Phytochemicals, which are predominantly found in plants, hold substantial medicinal value. Despite their potential, challenges such as poor oral bioavailability and instability in the gastrointestinal tract have limited their therapeutic use. Traditional intra/transdermal drug delivery systems offer some advantages over oral administration but still suffer from issues such as limited penetration depth, slow drug release rates, and inconsistent drug absorption. In contrast, microneedles (MNs) represent a significant advancement in intra/transdermal drug delivery by providing precise control over phytochemical delivery and enhanced penetration capabilities. By circumventing skin barriers, MNs directly access dermal layers rich in blood vessels and lymphatics, thus facilitating efficient phytochemical delivery. This review extensively discusses the obstacles of traditional oral delivery and the benefits of intra/transdermal delivery routes with a particular focus on the transformative potential of MNs for phytochemical delivery. This review explores the complexities of delivering phytochemicals through intra/transdermal routes, the development and types of MNs as innovative delivery tools, and the optimal design and properties of MNs for effective phytochemical delivery. Additionally, this review examines the versatile applications of MN-mediated phytochemical delivery, including its role in administering phytophotosensitizers for photodynamic therapy, and concludes with insights into relevant patents and future perspectives.

## 1 Introduction

Since ancient times, humans have relied on plants for sustenance and medicinal purposes. The emergence of phytochemistry in the 18th century marked a new era of scientific exploration into the chemical constituents of plants. Over subsequent centuries, advancements in technology have propelled research, leading to the discovery of numerous phytochemicals with significant pharmacological importance.^1^ Phytochemicals, alternatively known as phytoconstituents or herbal drugs, encompass the diverse array of primary and secondary metabolites found within plants.^2^ These compounds, characterized by their intricate chemical structures, are abundant in a wide range of fruits, vegetables, and herbal extracts.^3^ Phytochemicals have demonstrated efficacy in treating a myriad of disease conditions, such as cancer, psoriasis, arthritis, alopecia, dermatitis, obesity, and chronic wounds, demonstrating their versatility and potential in modern therapeutic interventions.^4^

The oral route is widely acknowledged as the primary means of delivering phytochemicals, benefiting from characteristics such as patient convenience, adherence, self-administration, and cost-effectiveness.^5−8^ In addition to their promising therapeutic potential, phytochemicals face challenges in achieving optimal oral bioavailability and bioactivity. These hurdles stem from their inherent characteristics, such as high lipophilicity, which restricts their solubility in aqueous environments. Additionally, they often exhibit poor stability within the harsh conditions of the gastrointestinal tract, hindering their absorption and subsequent entry into systemic circulation.^9^ Of these challenges, limited aqueous solubility stands out as a primary obstacle, as it necessitates the traversal of the unstirred water layer coating the epithelial surface for absorption to occur. Moreover, administering oral medications for skin conditions can increase systemic exposure, potentially causing systemic side effects, and may not adequately deliver therapeutic levels to the intended skin site.^10^ These drawbacks have spurred advancements in the intra/transdermal delivery of phytochemicals for treating both local and systemic conditions.

Intra/transdermal drug delivery (via gel, cream, ointment, or patch) is a promising approach for ensuring consistent drug levels and minimizing gastrointestinal side effects while offering both local and systemic treatment.^11^ Bypassing first-pass metabolism provides a convenient alternative to oral administration, enhancing bioavailability and therapeutic efficacy with prolonged therapeutic potential. This method allows therapeutic agents to permeate intact skin layers, enter the systemic circulation directly and reduce the risk of metabolic degradation associated with oral intake.^12^ To date, several phytochemical-based intra/transdermal formulations have been approved by regulatory authorities for numerous disease treatments. For example, scopolamine, first approved by the United States Food and Drug Administration (USFDA) on December 31, 1979, became available through Novartis’s Transderm Scop, a 2.5 cm^2^ transdermal patch that delivers 1.5 mg of scopolamine over 3 days. Scopolamine transdermal patches are used to prevent nausea, vomiting postanesthesia, surgery, and motion sickness.^13,14^ However, conventional intra/transdermal drug delivery systems encounter challenges due to restricted drug permeation from the skin barrier, absorption variability influenced by factors such as skin thickness and hydration, potential skin irritation or allergic reactions to adhesive components.^15^ Although intradermal delivery via conventional formulations to treat skin conditions has demonstrated efficacy, it remains suboptimal because of the presence of the outermost skin layer, the stratum corneum, which acts as a major barrier.^16^ This has paved the way for continued research into advanced intra/transdermal drug delivery systems.

Microneedles (MNs), also known as MN arrays or microarray patches, revolutionize intra/transdermal drug delivery by penetrating the surface of the skin, allowing compounds to reach the dermal layers rich in blood vessels and lymphatics for systemic absorption.^17−24^ Despite their ability to breach the skin barrier, MN insertion is generally associated with minimal discomfort because it involves targeting the superficial dermal layer with fewer pain receptors.^25,26^ This innovative approach offers precise delivery of both small and large molecules, facilitating localized or systemic therapeutic effects.^27,28^ Moreover, MNs offer distinct advantages over nanoparticles (NPs) incorporated into traditional dosage forms such as gels and creams in the context of transdermal drug delivery. MNs physically bypass the stratum corneum, which enables more efficient penetration of drugs into the deeper skin layers. In contrast, NPs rely on transcellular or trans-appendageal pathways to traverse the stratum corneum, thereby limiting their overall effectiveness. However, when combined with NPs, MNs not only improve skin penetration but also capitalize on the inherent benefits of NPs. These advantages include protecting the therapeutic payload from chemical or enzymatic degradation, enabling sustained release, and facilitating targeted drug delivery. This synergistic approach enhances the overall efficiency and effectiveness of dermal/transdermal treatments. To date, several studies have investigated the use of phytochemical-loaded MNs for treating various conditions.^29^

For example, one study conducted by our research group involved the development of curcumin (CUR) nanosuspension-loaded dissolving MNs for intradermal delivery.^30^ Another notable investigation by our group focused on the development of colchicine-loaded dissolving MNs for the management of gout.^31^ Additionally, we explored the utilization of carvacrol-loaded MN injection systems employing commercially available hollow MNs (AdminPen). Our comparative analysis involved the assessment of various hollow MNs, such as AdminPen 1500, AdminPen 777, and AdminPen 1200, and revealed that AdminPen 1500 demonstrated superior efficacy in managing polymicrobial biofilm-infected wounds.^32^ Furthermore, in another study, we engineered dissolving MNs loaded with artemether and lumefantrine for malaria treatment.^33^ Finally, we developed a hypericin-based photosensitizer-loaded in-house single hollow MN (1300 μm) for photodynamic therapy against skin cancer. The efficiency of this novel approach was compared with that of the commercially available AdminPen (1200 μm).^34^

**Figure 1:**
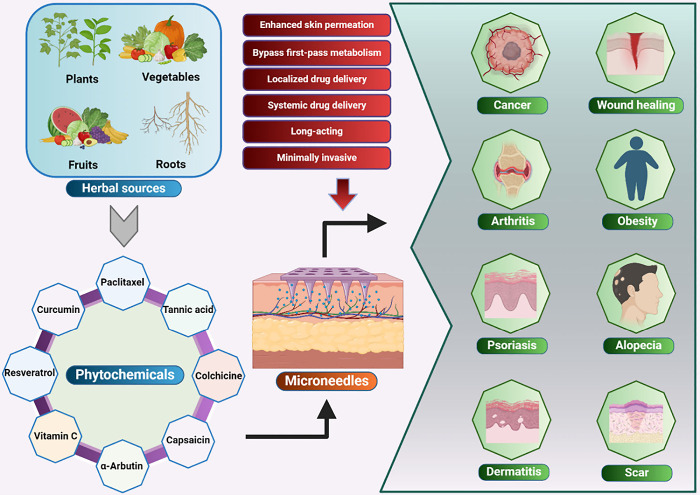
Schematic representation of MNs as an emerging platform for efficient intra/transdermal delivery of phytochemicals for the treatment of various ailments.

In this context, the present review offers a succinct exploration of the challenges associated with intra/transdermal drug/phytochemical delivery, as well as an examination of the emergence and diverse types of MNs. Additionally, this study delves into the advantages and optimal properties required for MNs to facilitate phytochemical delivery. This review further explores the various applications of MN-mediated phytochemical delivery, including its role in delivering phytophotosensitizers for photodynamic therapy (PDT) (Figure 1). Finally, this study highlights patents relevant to this field, providing a comprehensive overview of advancements in MN-mediated phytochemical delivery systems.

## 2 Challenges in Intra/Transdermal Phytochemical Delivery

The skin acts as the body’s primary shield against the external environment, covering an area of approximately 1.5–2.0 m^2^. It makes up approximately 15% of an adult’s total body weight. As the largest organ, its main job is to protect the body from physical, mechanical, and chemical assault from the outside. The skin is composed of three distinct layers: the epidermis, the dermis, and the hypodermis.^35^ Owing to the presence of blood capillaries within the dermis, the skin serves as the primary site for drug absorption, whether for localized or systemic delivery.^15^ However, the process of drug absorption through the skin presents significant challenges, primarily due to the initial barrier it encounters, known as the stratum corneum (SC). The SC is primarily composed of deceased keratinocytes, which, in conjunction with the ceramide lipid component, create a dense structure often likened to a brick-and-mortar arrangement.^11^

**Figure 2:**
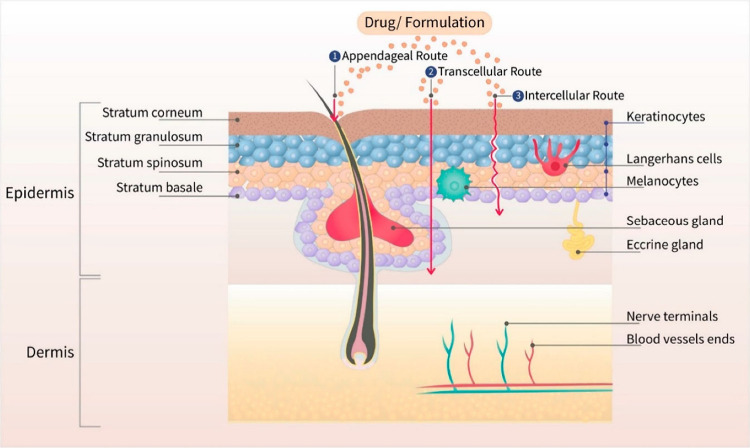
Various skin layers featuring primary pathways, such as the appendageal, transcellular (through the epidermis), and intracellular (across the epidermis), drug transport pathways. The outermost layer, known as the stratum corneum, serves as the primary obstacle for delivering drugs through the skin; this layer was reproduced from ref (36) licensed under CC BY 4.0. Copyright 2021 The Authors.

The “brick” element consists of keratin, a protein product derived from keratinocytes that varies from acidic to neutral in nature. Moreover, the “mortar” component is predominantly composed of lipids. These keratinocytes are interconnected through glycoprotein desmosomes known as corneodesmosomes.^27^ For drugs to enter the bloodstream, they must first traverse this intricate molecular arrangement within the skin. Drug absorption through the skin primarily occurs via transepidermal and transappendageal routes (Figure 2).^36^ In transepidermal absorption, drugs traverse lipid bilayers (transcellular) or intercellular (paracellular) spaces, with hydrophobic drugs favoring lipid membranes (transcellular) and hydrophilic drugs favoring intercellular pathways (paracellular). Compared with transepidermal pathways, transappendal absorption, which occurs through hair follicles or sweat glands, is vital for certain compounds but is constrained by smaller absorption areas.^11^ However, attaining successful drug permeation requires more than just possessing a balanced hydrophilic–lipophilic profile and an ideal partition coefficient, typically falling within the range of log P 2–3. Additional constraints are imposed by factors such as molecular weight (<500 Da) and the potential for ionization. For example, phenolic compounds derived from various plant sources present various physicochemical properties that affect skin permeation. Simple phenols such as caffeic acid, which have multiple ionizable groups (alcohol and carboxyl groups), are characterized by lower molecular weights and higher water solubilities and are thus limited by these groups. Moreover, flavonoid aglycons such as quercetin are highly hydrophobic, with exceptions such as catechins, and the glycosylation of compounds such as hesperidin leads to the formation of more hydrophilic derivatives, rendering them poorly permeable via the transdermal route.^37,38^ In this context, numerous researchers are actively working on enhancing the permeability of various synthetic drugs and phytochemicals through advanced intra/transdermal drug delivery systems. Over the past two decades, studies have shown that MNs can effectively address the aforementioned limitations by delivering a diverse range of drugs/phytochemicals directly to the viable epidermis or superficial layer of the dermis while piercing the epidermal layers in a painless manner. From there, they are subsequently transported to the systemic circulation via the dermal microcirculation. In the forthcoming sections, we looked into the emergence and various types of MNs, elucidating how they precisely deliver therapeutic agents intradermally or transdermally.

## 3 Emergence and Types of MNs

**Figure 3:**
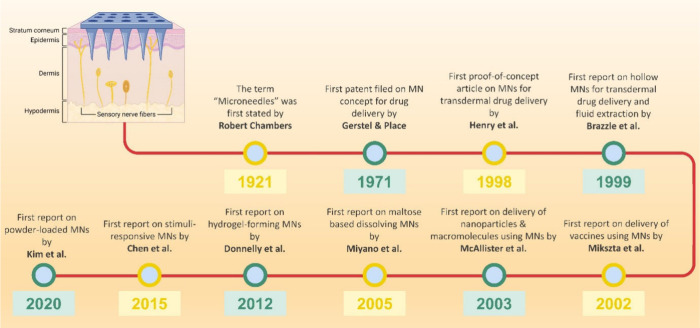
Timeline of the emergence of various types of MNs.

MNs, alternatively termed MN arrays or microarray patches (MAPs), represent an innovative system resembling a patch but featuring miniature projections perpendicular to its surface (base plate).^39−41^ After application to the skin, the microprojections of MNs breach the epidermal barrier, facilitating the delivery or deposition of compounds into the underlying dermis.^42^ This tissue layer harbors a rich vascular and lymphatic network, providing a favorable environment for systemic absorption of the drug.^43−45^ While MNs disrupt the skin barrier to deliver drugs, pain from their insertion is typically minimal. This is because the MNs penetrate the superficial dermal layer, where fewer pain receptors are present.^46^ With respect to the genesis of MNs, the term “microneedles” was initially coined by Robert Chambers in 1921. Chambers devised a glass-based MN designed for microdissection purposes.^47^ From 1921 to 1970, there were no notable advancements in the field of MNs. However, in 1971, Gerstel and Place introduced a strategy for delivering drugs using both solid and hollow MNs and subsequently filed a US patent for their invention.^48^ After a 25-year lag, in 1998, Henry and colleagues published a pioneering study on the transdermal delivery of MN.^49^ Brazzle and collaborators developed the first hollow MNs in 1999 for transdermally delivering drugs and extracting interstitial fluid from the skin.^50^ In 2002, Mikszta et al. first delivered vaccines via silicone MNs.^51^ In 2003, McAllister and colleagues were the first to report the transdermal delivery of nanoparticles and macromolecules with the aid of both solid and hollow MNs.^52^ In 2005, Miyano and co-workers introduced dissolvable MNs prepared using maltose for the first time.^53^ Significant development occurred in 2012 when a distinct type of swellable polymeric MN, named “hydrogel-forming MN,” was first introduced by Donnelly and colleagues (Figure 3).^54^ In 2015, Chen and colleagues, for the first time, developed stimuli-responsive, near-infrared (NIR) light-activated microneedles for small-molecule delivery, utilizing polycaprolactone microneedles loaded with silica-coated lanthanum hexaboride (LaB6@SiO2) nanostructures.^55^ In 2020, Kim and colleagues were the first to report the use of powder-loaded MNs for transdermal delivery of high-dose insulin, demonstrating improved efficacy.^56^

**Figure 4:**
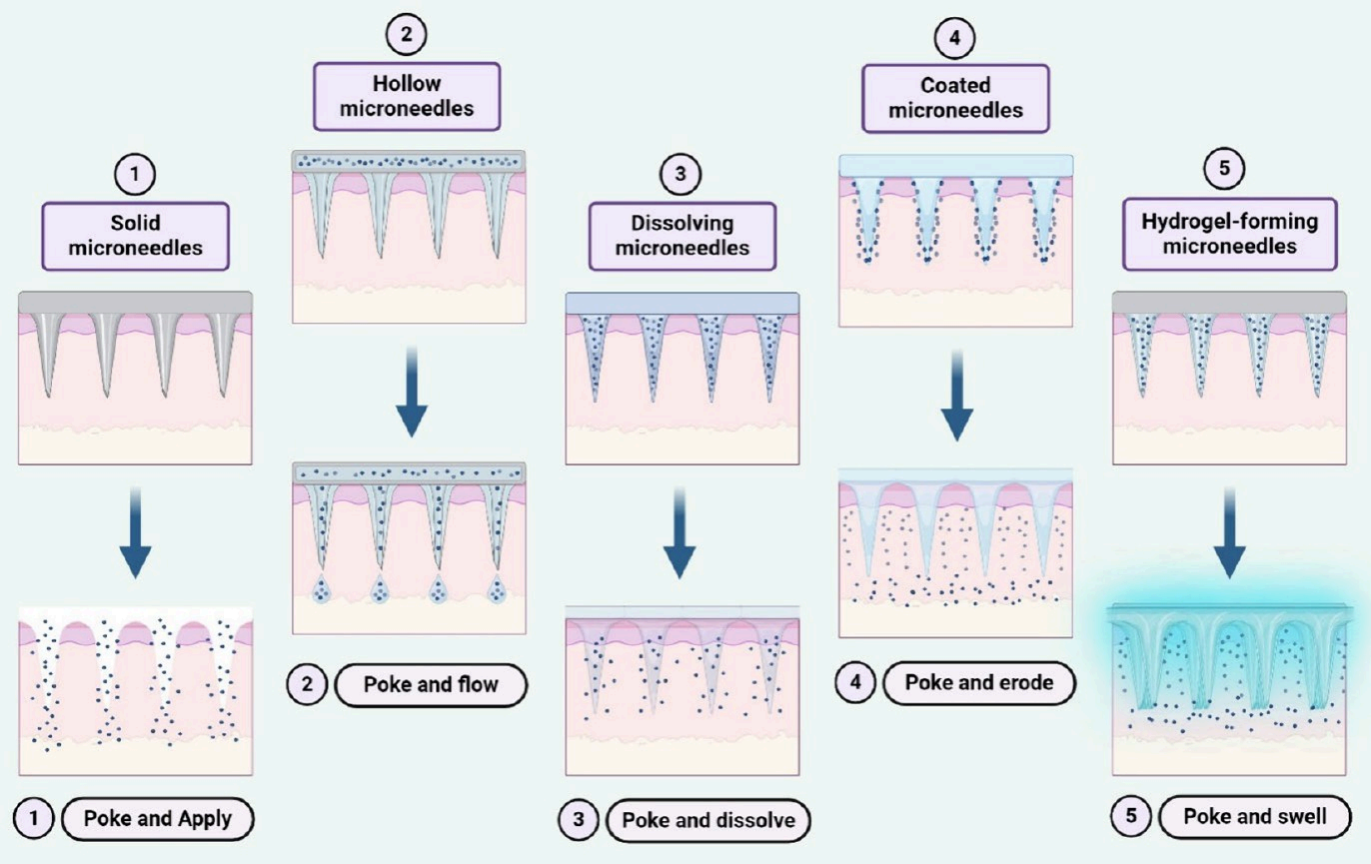
Various types of MNs are utilized to enhance intradermal drug delivery through different mechanisms: (1) Solid MNs pierce the skin and are then removed, allowing the application of a drug or nanoparticle formulation. (2) Hollow MNs pierce the skin, enabling liquid drug release through active infusion or diffusion. (3) Dissolving MNs rapidly or slowly release drugs incorporated within them. (4) Coated MNs coated with drugs undergo erosion of the drug-coated layer and deliver the contents. (5) Hydrogel-forming MNs absorb tissue fluids, promoting drug diffusion from a patch through swollen microprojections.

MNs are classified into different categories: solid, coated, hollow, dissolving, and hydrogel-forming.^57^ Solid MNs operate on the “poke and apply” principle, where they are inserted onto the skin to create microsized conduits. These micropores serve as channels for the delivery of various drugs through traditional formulations, such as ointments, gels, lotions, patches, and creams.^58^ Hollow MNs follow the “poke and flow” concept and consist of multiple hole-consisting MNs attached to a syringe, resembling a hypodermic needle with a syringe.^59^ The medication solution dispensed by these MNs is administered transdermally or intradermally, akin to the conventional delivery method of needle-based syringes, which dispenses medications subcutaneously, intramuscularly, or intravenously.^59^ Coated MNs adopt the “poke and erode” principle to deliver drugs into the dermis or deeper layers. These MNs are coated with therapeutic agents, applied to the skin, and undergo microdissolution (erosion) of the coated drug in the skin interstitial fluid for drug delivery.^60,61^ Dissolving MNs adhere to the concept of “poke and dissolve,” wherein the MNs loaded with medication are inserted into the skin to dissolve entirely in the interstitial fluid, thereby releasing the medication.^62−64^ Finally, hydrogel-forming MNs operate according to the “poke and swell” principle. In the presence of interstitial fluid, these MNs can expand, facilitating medication delivery through diffusion from needles without dissolving, and they can be effortlessly removed from the skin intact after drug release is complete (Figure 4).^65−69^

A study conducted by Aung and colleagues examined the differences in drug delivery efficiency and bioavailability between dissolving MNs (DMNs) and hydrogel-forming MNs (HMNs).^70^ DMNs, which dissolve fully upon application, allow quicker and more efficient drug release into the skin. In contrast, HMNs work by absorbing interstitial fluid and gradually swell, resulting in a slower release of the drug. Research has shown that DMNs are much more effective at delivering α-arbutin, achieving a permeability rate that is 4.5 times greater than that of HMNs. Additionally, DMNs reached a greater peak intradermal concentration of 5.33 μg/mL than did HMNs, which delivered a lower concentration of 1.47 μg/mL, emphasizing the superior delivery ability of DMNs.

In recent years, stimuli-responsive MNs have made significant advancements in intra/transdermal drug delivery over traditional MNs.^25^ These innovative stimuli-responsive MNs are engineered to react to various internal or external triggers, such as changes in pH, temperature, or exposure to light, allowing for the precise, on-demand release of medications. This tailored approach reduces the likelihood of overdosing and enhances the accuracy of treatment. In contrast, conventional MNs, though effective for delivering drugs through the skin, typically release their contents immediately/over a period of use without adapting to the body’s fluctuating needs (releasing insulin only when there are high blood glucose levels).^25^

## 4 Ideal Design and Properties of MNs for Phytochemical Delivery

MNs are designed primarily to penetrate the skin effectively while maintaining structural integrity. An ideal MN design balances a low insertion force with a high fracture resistance.^39,71^ For hydrophilic drugs, MNs need only breach the outer skin layer to facilitate transdermal delivery, allowing the drug in the MN base plate to diffuse through microchannels. However, the delivery of hydrophobic drugs to the skin requires MNs to prevent the waste of any drug particles. Thus, an effective MN system necessitates careful consideration of the geometry, mechanical properties, and drug loading to optimize delivery efficiency. Layering MNs ensures concentrated drug loading in the inserted part of the projection, minimizing waste and maximizing delivery efficiency.^72^ Among others, dissolving and hydrogel MNs made of various polymers are most widely preferred for their efficacy in delivering drugs. Thus, further discussions will focus on dissolving and hydrogel MNs.

The insertion capability, drug loading capacity, and mechanical properties of MNs are intricately determined by their geometric characteristics, which include parameters such as the MN tip radius, height (H), base width (W), aspect ratio (AR), interspacing (IS), and shape. Furthermore, the choice of materials for MN fabrication and the properties of the skin significantly influence their performance. The natural elasticity of the skin presents a significant obstacle to the consistent penetration of MNs. Research suggests that, depending on the length of the MNs, the skin may fold around them, resulting in partial or incomplete piercing of the stratum corneum.^73^ In the literature, the prevailing shapes for MN design are conical or pyramidal, typically featuring heights ranging from 50 to 2000 μm, base widths ranging from 50 to 500 μm, and interspaces ranging from 50 to 600 μm. Meticulous selection of these parameters is imperative for generating MNs with optimal functionality, ensuring maximal mechanical robustness, insertion capacity, and drug loading, thereby bolstering the efficiency of drug delivery systems.^71,74^

Studies have shown that MNs with pyramid shapes tend to have superior mechanical strength compared with those with conical shapes, which is attributed to their broader base and larger cross-sectional area. This design reduces the risk of needle bending or breakage during skin penetration, making pyramid-shaped MNs more reliable than conical-shaped MNs for intra/transdermal drug delivery.^72^ Furthermore, the aspect ratio and shape of MNs are critical parameters influencing their mechanical properties. To increase drug loading per unit area, options include increasing MN density or reducing interspacing, as well as increasing needle length and width. However, excessively widening needle bases to reduce the aspect ratio or MN density can impede efficient skin insertion. For example, a study demonstrated that MN arrays with higher density and smaller interspacing had a lower insertion capability than did arrays with larger MNs. Similarly, an excessive increase in needle length to increase the aspect ratio beyond 2 may compromise MN mechanical strength.^74,75^

To increase MN drug delivery efficiency and insertion capability, strategies include enhancing the needle height, reducing the needle tip radius, or adjusting the interspacing.^71,76,77^ Sharper MN tips facilitate easier skin puncturing, as demonstrated by chitosan MNs with a 5 μm tip radius achieving 2-fold greater insertion depth than those with a 10 μm tip radius. Pyramidal polymeric MNs with smaller aspect ratios and sharper tips are particularly effective for skin insertion.^78^ Generally, an optimal MN width of 300 μm and interspacing are recommended for achieving both effective insertion and reasonable drug loading per unit area, with an ideal aspect ratio of 2 for optimal mechanical strength.^44,74^

On the basis of the studies discussed above, ideal MNs for delivering phytochemicals should possess several key characteristics. First, they should be layered, with phytochemicals loaded exclusively in the MN projections, particularly in the inserted part, to minimize any drug waste. Additionally, these MNs should be crafted from robust materials to ensure that they possess sufficient mechanical properties for effective insertion. Importantly, they are composed of biocompatible or biodegradable polymers with molecular weights less than 60,000 Da, facilitating rapid clearance from the body after deposition.^79^ MNs of an appropriate length, less than 1 mm, are preferable for ensuring transdermal phytochemical delivery without causing discomfort. Moreover, they should be designed to be applied to specific anatomical locations within the body and exhibit flexibility to conform to skin contours. Low insertion forces and high break forces are also desirable attributes. Most importantly, the MN geometry must be optimized to accommodate skin deformation, allowing for maximum insertion capability and high phytochemical loading per unit area. Finally, MNs featuring 800–1000 μm projections, an aspect ratio of 2–3, and an interspacing of approximately 300 μm offer one potential option. Alternatively, MNs with pedestal-like structures of similar geometry can mitigate mechanical concerns.^39,72^

## 5 Applications of MN-Mediated Phytochemical Delivery

**Figure 5:**
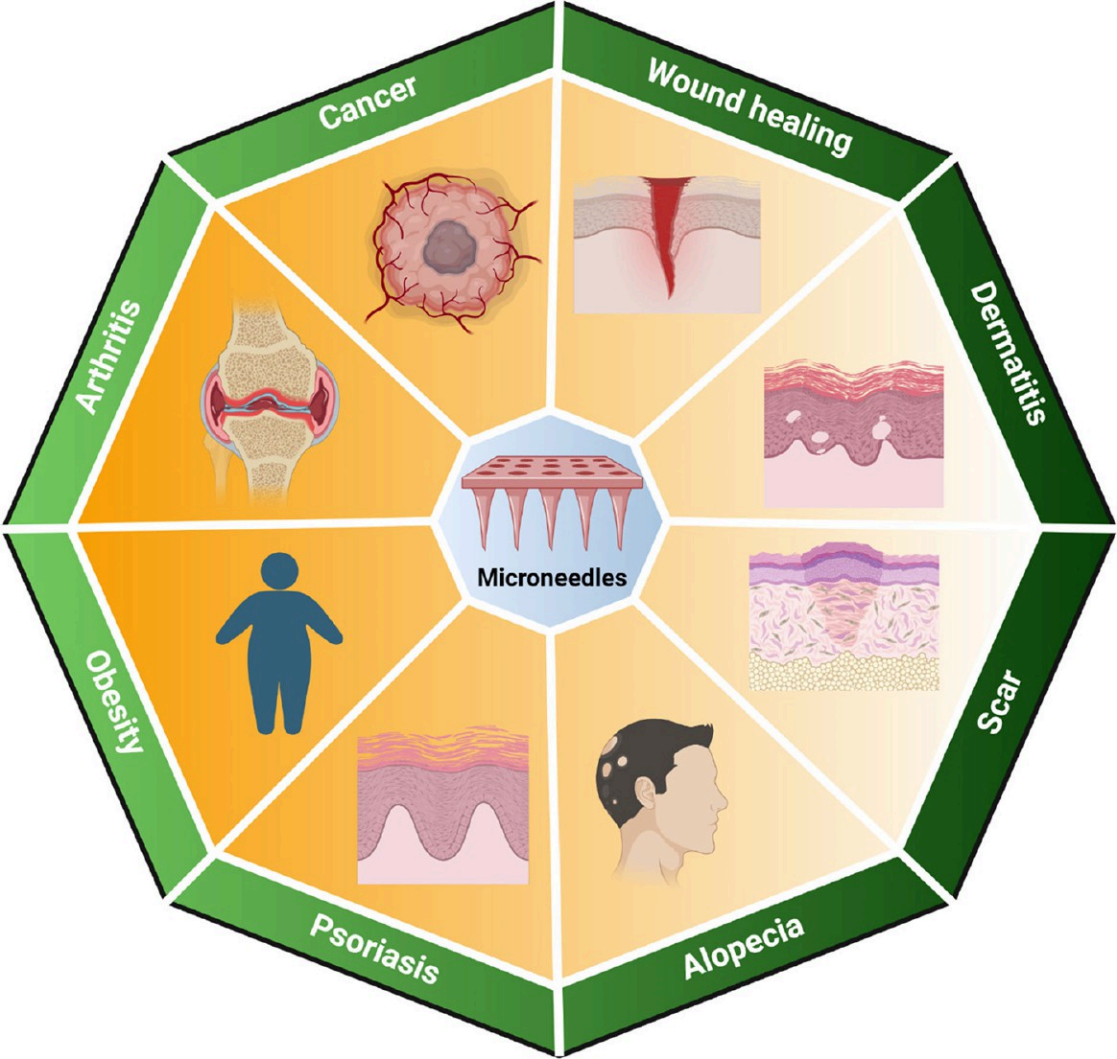
Illustration of the use of phytochemical-loaded MNs for treating a variety of conditions.

MN-based drug delivery systems offer a promising approach for the intra/transdermal delivery of phytochemicals, providing several advantages over conventional formulations (gels, creams, ointments, and patches).^29^ By penetrating the skin’s barrier, MNs enable enhanced permeation of phytochemicals into the dermal layers, ensuring consistent and predictable delivery while minimizing patient discomfort. This precise delivery mechanism allows for the controlled release of phytochemicals, optimizing therapeutic outcomes and increasing their bioavailability through direct access to the dermal vascular network. Moreover, MNs offer prolonged therapeutic potential with sustained release profiles, reducing the frequency of administration and improving patient compliance.^80^ Owing to their versatility in accommodating different formulations and dosages, MNs present a flexible platform for the delivery of phytochemicals across various medical applications. Compared with oral administration, MN-mediated intra/transdermal delivery bypasses first-pass metabolism, aiding in achieving significant effects at lower doses. Additionally, MNs enhance safety by minimizing systemic side effects while treating local skin conditions.^81^ Nevertheless, the rapid onset of action facilitated by MNs enables quicker therapeutic effects than oral administration. On the other hand, MNs enhance the permeability of the skin to phytochemicals, subsequently reducing the risk of skin irritation or allergic reactions associated with conventional transdermal patches.^29,57,82^ Finally, MNs can facilitate combination therapy by delivering multiple phytochemicals or other therapeutic agents simultaneously, resulting in synergistic effects. The subsequent sections intricately explore the diverse applications of MN-mediated delivery of phytochemicals (Figure 5).

### 5.1 Cancer

Cancer, a pervasive global health concern affecting diverse populations, poses significant challenges for low- and middle-income countries.^83^ The anticipated doubling of the global cancer burden to 29–37 million cases by 2040, particularly impacting lower-middle-income nations, underscores its status as a leading cause of nearly 10 million deaths worldwide in 2020.^84^ Superficial skin tumors such as melanoma, squamous or basal cell carcinoma, breast cancer, oral epidermoid cancer, and head and neck cancer not only pose a physical threat but also take a toll on patients’ mental well-being.^85^ Chemotherapeutic agents encounter numerous obstacles, such as multidrug resistance, elevated toxicity, imprecise delivery to targeted areas, and the necessity for frequent high-dose administration.^86−94^ These challenges collectively pose formidable barriers throughout the course of therapy.^95^ Over the past 60 years, numerous phytochemicals derived from plants, including alkaloids, phenolic compounds, terpenes, and sulfur-containing compounds, have undergone thorough exploration for their potential in combating cancer.^96−98^ Some of the commonly used phytochemicals for cancer treatment include paclitaxel (PTX), vincristine, curcumin (CUR), resveratrol (RSV), quercetin, etc. Regrettably, these treatment approaches result in minimal curative outcomes. This is attributed primarily to the significant drawbacks of conventional formulations employed for phytochemical delivery, inadequate solubility, low skin permeability, and poor pharmacokinetic characteristics.^99^ These factors present substantial obstacles to achieving effective therapeutic interventions.^100,101^ Thus, research has focused on superficial skin tumor treatment trends toward the utilization of advanced transdermal drug delivery.^102,103^ MNs are characterized as distinctive patches featuring minute projections affixed to one surface of a supporting base or patch. In contrast to conventional transdermal drug delivery systems, MNs pierce the stratum corneum and deliver the drug directly into either the viable epidermis or superficial layer of the dermis.^27^

CUR, derived from *Curcuma longa*, has robust anticancer effects. Its mechanisms involve hindering cellular growth, initiating autophagy, augmenting reactive oxygen species, stimulating ferroptosis, curtailing invasion, and encouraging programmed cell death in diverse cancer varieties.^104,105^ Studies have also shown that the inhibition of the JAK-2/STAT-3 signaling pathway, nuclear factor kappa B, etc., is among the main mechanisms responsible for the antimelanoma activity of CUR, making these compounds potential candidates for the treatment of melanoma.^106^ A recent study by Zhou and colleagues fabricated CUR-loaded β-cyclodextrin-attached gelatine methacryloyl-based MNs (CUR@β-CD-GelMA MNs) for the treatment of melanoma.^107^ The primary rationale for incorporating β-CD is to increase the solubility and stability of CUR through the formation of an inclusion complex. Here, the authors developed an 11 × 11 array of MN array patches with heights and base widths of 600 and 300 μm, respectively, via the micromolding technique. The MNs were then cross-linked with UV light for a maximum duration of up to 30 s. However, the authors did not report the amount of CUR loaded into the tips of the MN array patch. Since CUR is light sensitive, the authors have also not provided any details on the stability of CUR post-UV light irradiation. Furthermore, the authors illustrated that the manipulation of the cross-linking time in MN arrays allows for the modulation of the mechanical properties and drug release characteristics. They reported that extended cross-linking times result in a higher density of cross-linked networks, requiring increased force to achieve comparable displacement. Furthermore, 24 h of monitoring in vitro CUR release from the MNs revealed that prolonged cross-linking extended the duration of drug release. The MNs without cross-linking released 90% of the CUR after an 8 h incubation, whereas the MNs cross-linked for 30 s released only approximately 50% after a 24 h incubation. Finally, the authors conducted an antimelanoma study using a 3D skin cancer model (3D B16F10 spheroids). The formed spheroids had a diameter of 400 μm and a thickness of 1 mm. With a needle height of 600 μm, the MNs effectively penetrated the extracellular matrix, enhancing CUR delivery to the target site. The MN array patch exhibited greater efficacy against melanoma than did the flat patch, primarily because of its ability to penetrate deeper into the spheroid model, which was confirmed by a live-dead assay after 24 h. However, assessing the CUR deposited in the 3D spheroid model would provide much more insight into the concentration of CUR required to reduce specific-sized 3D B16F10 spheroids. Overall, CUR@β-CD-GelMA MNs exhibit promising attributes, positioning them as prospective candidates for the treatment of melanoma.

**Figure 6:**
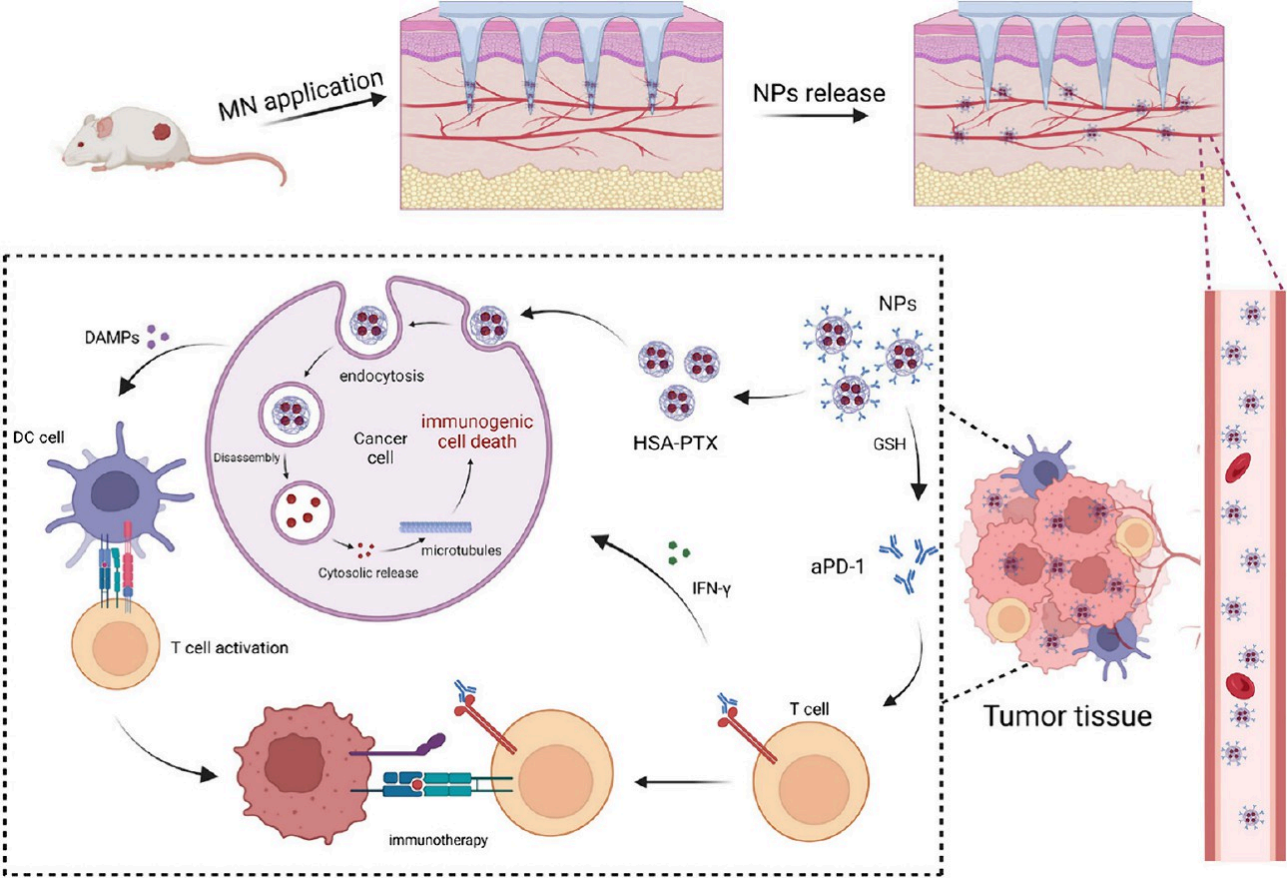
Schematic illustration of the synergistic codelivery of aPD-1 and PTX to MNs for chemotherapy and immunotherapy. Reproduced with permission from ref (110). Copyright 2024 Wiley.

PTX, a potent natural anticancer agent belonging to the diterpene class of phytochemicals, is extracted primarily from the bark of the yew tree *Taxus brevifolia Nutt*. It was discovered in the 1960s by Dr. J.L. Hartwell from the National Cancer Institute while screening antitumor agents in the plant kingdom.^108^ Furthermore, chemoimmunotherapy is a new way to treat cancer that integrates regular chemotherapy with immunotherapy. The goal is to increase the effectiveness of cancer therapy by using chemotherapy to attack cancer cells and immunotherapy to increase the ability of the immune system to fight against cancer at the same time.^109^ In this context, Wang and teammates developed PTX and anti-PD-1 (aPD) monoclonal antibody-loaded dissolving MN array patches (aPD/PTX@NP@MNs) for the treatment of triple-negative breast cancer (TNBC).^110^ Here, the authors used nanoparticle albumin-bound PTX (PTX@NPs). When these PTX@NPs are mixed with aPD, the albumin present in the PTX@NPs forms a dual-component system by establishing disulfide bonds at a 1:1 molar ratio between the Fab and Fc fragments of IgG, resulting in the creation of aPD/PTX nanoparticles (aPD/PTX@NPs). Furthermore, the aPD/PTX@NPs were incorporated into a hyaluronic acid (HA)-based MN array patch with a 12 × 12 array, featuring a height of 1200 μm and a base width of 300 μm. The MNs exhibited a combination of tetrahedral and pyramidal structures, resulting in the loading of approximately 150 μg of aPD/PTX NPs. Moreover, an in vivo study conducted in a murine model of breast cancer (4T1) revealed that administering aPD/PTX@NPs via MNs resulted in more effective therapeutic outcomes than intratumoral injections did. Additionally, aPD/PTX@NP@MNs not only promoted immunogenic cell death in tumor cells, enhancing the infiltration of cytotoxic T cells into the tumor but also hindered immune checkpoints on T cells, relieving the suppression of cytotoxic T-cell activity and leading to a synergistic effect in TNBC (Figure 6).

### 5.2 Wound Healing

Wound emergence is a result of either the sequential loss of function in any skin layer or the impact of internal and external factors, leading to a disturbance in the histological composition of the skin tissue.^111,112^ Plant-derived constituents have emerged as potent therapeutics for wound management.^113−117^ These alternatives, recognized for their safety, reduced toxicity, and cost-effectiveness, play crucial roles in promoting wound repair by targeting several signaling pathways, including the nuclear factor kappa B (NFκB), Jun N-terminal kinase (JNK), nuclear factor erythroid 2–related factor 2 (Nrf2), and extracellular signal-regulated kinase (ERK) pathways. Phytochemicals scavenge reactive oxygen species (ROS), demonstrating antibacterial efficacy, exerting antioxidant effects, and eliciting anti-inflammatory effects. Collectively, these attributes play pivotal roles in fostering an effective wound healing process.^118^ Over the past decade, the use of MNs loaded with various phytochemicals for the effective management of wounds has been reported. In contrast to conventional dressing systems, MNs aim to increase therapeutic availability in the wound bed by tightly attaching to it, ensuring controlled spatial distribution through the management of the drug content of individual needles. Furthermore, the approaches utilized by MNs to deliver therapeutics to the wound bed can be categorized into active, passive, and stimuli-responsive release strategies, depending on the extent of control over the temporal release profile.^119−121^

**Figure 7:**
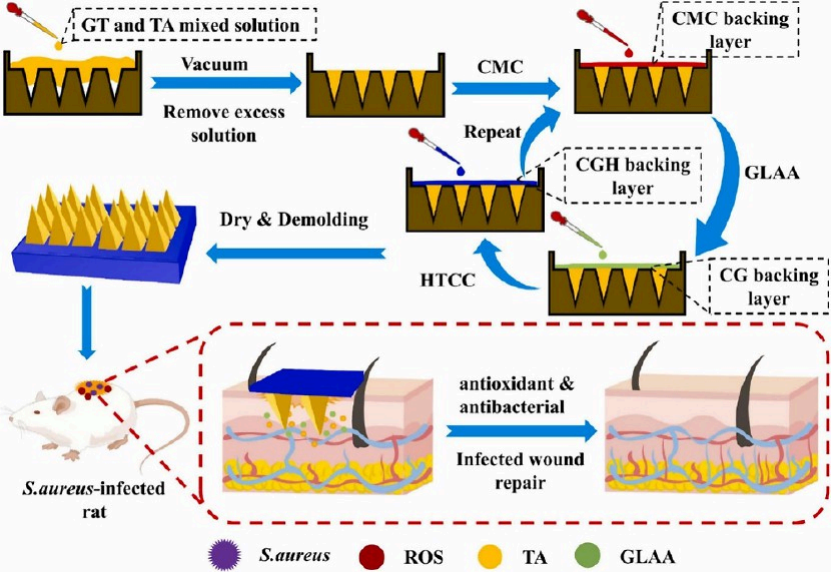
Illustration depicting the manufacturing process of antibiotic-free antibacterial and antioxidant agents. The CGH/GTA MN patch, along with accelerated *S. aureus*-infected full-thickness wound healing, functions by eradicating *S. aureus* infection and suppressing ROS production Reproduced with permission from ref (127). Copyright 2023 Elsevier

Tannic acid (TA), characterized as a polyphenolic compound, possesses antibacterial, anti-inflammatory, and antioxidant properties due to its unique catechol structure.^122−124^ Several investigations, including both in vitro and in vivo studies, have consistently shown the remarkable capacity of TAs to promote wound healing. Its ability to stimulate the wound site has been well documented, triggering the release of crucial growth factors. Furthermore, TA has notable effectiveness in anti-inflammatory processes, further enhancing its role in the healing cascade. Notably, its impact on the secretion of growth factors has been particularly emphasized, with a key focus on activating the Erk 1/2 pathway.^125,126^ Therefore, Li and colleagues developed a TA-loaded gelatin MN array patch via a micromold technique by applying a vacuum, with the baseplate exclusively prepared using carboxymethyl cellulose, 2-O-α-D-glucopyranosyl-L-ascorbic acid, and chitosan (Figure 7).^127^ The ascorbic acid (AA) and chitosan present in the base plate also exhibit antioxidant and antibacterial effects, further enhancing the wound healing process. With a 20 × 20 array and a pyramid-shaped needle structure, the height and base width of the developed MN patch were 770 and 410 μm, respectively. The developed MNs effectively penetrated the skin, showing pH-responsive release properties for both TA and AA. Furthermore, the MNs demonstrated excellent antioxidative properties, exhibiting more than 80% DPPH scavenging efficacy and 100% ABTS efficiency. Additionally, the MNs displayed 100% antibacterial activity against *Escherichia coli* and *Staphylococcus aureus*. Nevertheless, in a rat model of *S. aureus-*infected wounds, the MN array patch not only efficiently accelerated the healing process but also effectively inhibited *S. aureus* infection, promoted angiogenesis, suppressed inflammation, and accelerated regeneration. Overall, this MN patch shows promise as a potential candidate for effectively treating infected wounds.

Multiple investigations have demonstrated that the potential of CUR to improve wound healing stems from its diverse biochemical effects. These properties include antioxidant, anti-inflammatory, and antimicrobial properties.^128−130^ In addition to these attributes, CUR plays a pivotal role in the healing of cutaneous wounds by influencing tissue remodelling, facilitating the formation of granulation tissue, and promoting the deposition of collagen. Studies have underscored its beneficial impact on wound healing, demonstrating improvements in heightened vascular density, increased proliferation of fibroblasts, and epithelial regeneration.^131,132^ Thus, Hasnain and Teammates developed a CUR-loaded chitosan/polyvinyl alcohol-based hydrogel MN array patch (CUR@CH/PVA MNs) for the effective management of wounds.^133^ The developed 15 × 15 pyramidal MNs, with a height of 345 μm and a base width of 250 μm, exhibited remarkable swelling properties, facilitating the absorption of tissue fluid from the wound and thereby expediting wound closure. The amount of CUR loaded into the MN patch with 225 needles was determined to be 122 μg. Moreover, the MNs demonstrated a controlled release profile characterized by a gradual increase in drug release, reaching 56% at 24 h and achieving a maximum of 70% after 48 h. Finally, in an in vivo study involving male Wistar rats, wound healing progression was monitored over a 14-day period using both blank and CUR@CH/PVA MNs. Accelerated wound closure of 70% by day 14 was observed for the CUR@CH/PVA MNs, surpassing the 52% and 37% closure rates in the blank MNs and control groups, respectively, indicating that CUR@CH/PVA MNs are potential candidates for efficient wound healing. While the authors have evaluated the antibacterial potential of MNs separately in this study, notably, an infectious wound animal model was not employed in the investigation.

### 5.3 Arthritis

Arthritis, defined as a distinct manifestation, is characterized by pronounced joint inflammation.^134^ There are several types of arthritis, the most common of which are rheumatoid arthritis (RA), gout, psoriasis, and osteoarthritis. Contemporary therapeutic strategies for RA, including disease-modifying antirheumatic drugs (DMARDs), glucocorticoids (GCs), nonsteroidal anti-inflammatory drugs (NSAIDs), etc., strive to mitigate joint inflammation and pain.^135^ The American College of Rheumatology strongly emphasizes the limited effectiveness and elevated toxicity of current medications for treating RA. Consequently, there is widespread dissatisfaction among patients, leading to 60–90% of individuals with RA turning to herbal-based complementary and alternative medicine.^136^

The administration of medications for the treatment of RA involves either oral ingestion or direct injection into the affected joints (intra-articular).^137^ However, the oral administration of drugs is limited by their limited bioavailability and increased side effects, curtailing their widespread clinical adoption. Furthermore, opting for injections into the joint cavity, although effective, introduces challenges such as pain, inconvenience, and the potential for infection. These factors collectively amplify the psychological strain experienced by patients dealing with RA. To address this challenge, the utilization of MNs has emerged as an advanced transdermal drug delivery system. The MNs facilitate direct drug delivery to the joint cavity via the joint skin, enabling targeted concentration of the drug at the affected site. This approach not only circumvents the systemic side effects associated with traditional drug delivery methods but also enhances the overall bioavailability of the drug, presenting a promising solution to the prevailing issue.^138,139^

Tetrandrine, derived from *Stephania tetrandra S*., is an alkaloid belonging to the bisbenzylisoquinoline (BBI) class. It has been shown to mitigate RA by hindering the inflammatory response of macrophages, curbing excessive fibroblast proliferation, and suppressing the formation of pannus.^140^ Both oral and injectable tetrandrine are available on the market. Nevertheless, its limited water solubility results in diminished oral bioavailability and presents challenges in terms of injection-based administration. To overcome this issue, Hu and teammates developed tetrandrine-encapsulated PLGA NP-loaded dissolving MN array patches for treating RA.^141^ With a 15 × 15 array and a height of 500 μm, the developed peach gum polysaccharide-based MNs were loaded with 1.5% w/w tetrandrine. Furthermore, the developed MNs significantly increased the absorption of tetrandrine through the transdermal pathway, and this effect was amplified by incorporating stealth mechanisms to evade phagocytes and leveraging inflammatory acidity for triggered release. The observed amelioration of adjuvant-induced arthritis demonstrated more robust control of the STAT3/p-STAT3 and JAK2/p-JAK2 pathways, indicating that these pathways hold significant promise as therapeutic strategies for the management of RA.

**Figure 8:**
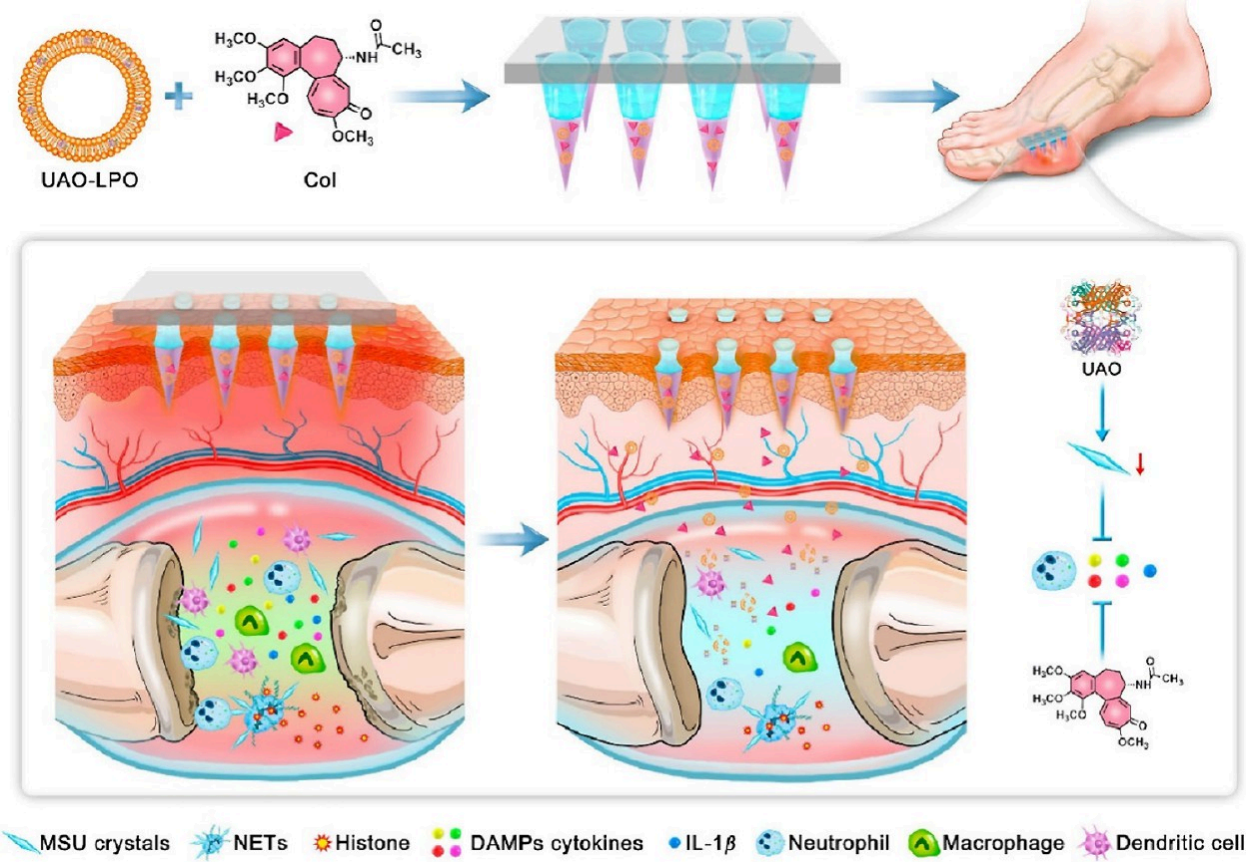
Illustration outlining the preparation of UAO-LPO/Col-MNs and their application in managing gout. The MNs are composed of three layers: a needle tip layer, a separation layer, and a base layer. UAO-LPO and Col were coloaded into the chitosan-containing needle tip layer. Upon topical administration of UAO-LPO/Col-MNs to gouty joints, the separation layer dissolves, leaving the needle tip layer in the skin to release Col and UAO-LPO continuously. UAO-LPO enters the joint cavity, which releases UAO to catalyze the oxidation of MSU and reduce MSU crystals. Col exhibits potent anti-inflammatory effects by inhibiting neutrophil recruitment, thereby reducing the release of damage-associated molecular pattern (DAMP) cytokines and blocking the NLRP3/IL-1β inflammasome activation induced by MSU crystals. Colchicine and uricase collaborate for effective gout management. Reproduced from ref (144), licensed under CC BY 4.0. Copyright 2023 Chinese Pharmaceutical Association and Institute of Materia Medica, Chinese Academy of Medical Sciences.

Gout is the prevailing form of inflammatory arthritis, stemming from the accumulation of monosodium urate crystals in and around structures adjacent to joints.^142^ It is typically addressed through the administration of anti-inflammatory medications. Colchicine, an herbal pharmaceutical agent originating from *Colchicum autumnale*, has received regulatory approval from the FDA for its efficacy in both the prophylaxis of gout and the management of acute gouty flares as a first-line therapy via oral administration.^143^ However, the effective management of gout flares with colchicine necessitates precise dosage control, given the unclear boundaries between therapeutic, toxic, and lethal doses within its narrow therapeutic range. Thus, Yang and colleagues developed colchicine and uricase liposome-loaded chitosan-based separable dissolving MNs for prolonged management of gout (Figure 8).^144^ The developed 12 × 12 array MNs were 800 μm in height and 300 μm in base width. Furthermore, the developed MNs without chitosan demonstrated a gradual increase in permeation, reaching 57% at 2 h and 71% within 48 h. In comparison, 71% of the MNs developed with chitosan were permeable over a 7-day period, highlighting the considerable influence of chitosan on drug penetration dynamics. Finally, the developed MNs displayed efficacy in preventing gout flares in an acute gout model and exhibited noteworthy effectiveness in minimizing bone damage caused by MSU crystals in a chronic gout model, making them promising options for managing gout.

### 5.4 Psoriasis

Psoriasis is a widespread skin condition rooted in immune-mediated inflammation and is characterized by expedited epidermal differentiation and proliferation.^145^ Approximately 125 million individuals worldwide suffer from psoriasis, constituting an estimated 2–3% of the total population worldwide.^146−150^ Betamethasone dipropionate, clobetasol propionate, amcinonide, hydrocortisone, tacrolimus, methotrexate, apremilast, cyclosporine, and other topical and systemic drugs are currently used for the management of psoriasis.^151^ Although these treatments offer relief from psoriasis symptoms, a complete cure remains elusive. Additionally, these therapies often have a range of side effects.^152^ In the pursuit of alternative treatments, considerable focus has shifted toward natural compounds derived from plants owing to their safety profile and widespread availability. Numerous studies have demonstrated the efficacy of specific phytochemicals, such as CUR, genistein, and RSV, in mitigating psoriasis. These compounds operate through diverse molecular mechanisms, including the induction of apoptosis, inhibition of angiogenesis, and suppression of inflammation arising from reactive oxygen species and the overexpression of inducible NO synthases.^153,154^ Topical therapy encompasses the application of medications directly to the affected skin, utilizing various dosage forms such as ointments, creams, gels, etc. However, it suffers from poor skin permeability, a sticky and greasy nature of vehicles, a lack of long-term controlled release, etc. To circumvent these challenges, researchers have devised an MN-based dermal drug delivery system to deliver antipsoriatic drugs precisely to the targeted site.^145^

Epigallocatechin 3-gallate (EGCG) is the predominant and most potent catechin found in green tea. Its application in treating psoriasis involves the elimination of reactive oxygen species (ROS), serving as a targeted approach to counteract the oxidative stress associated with this skin condition. However, it suffers from poor bioavailability. Therefore, Jia and colleagues fabricated PVA-based MN arrays loaded with EGCG and dexamethasone.^155^ This innovation aims to synergistically rectify the imbalance in subcutaneous immune homeostasis as a therapeutic intervention for psoriasis. The developed MNs successfully alleviated symptoms in imiquimod-induced psoriatic mice. This involves a decrease in epidermal thickness, restoration of the immune microenvironment balance of the skin, correction of immune cell disorders, and attenuation of hyperproliferation of keratinocytes within psoriatic lesions.

**Figure 9:**
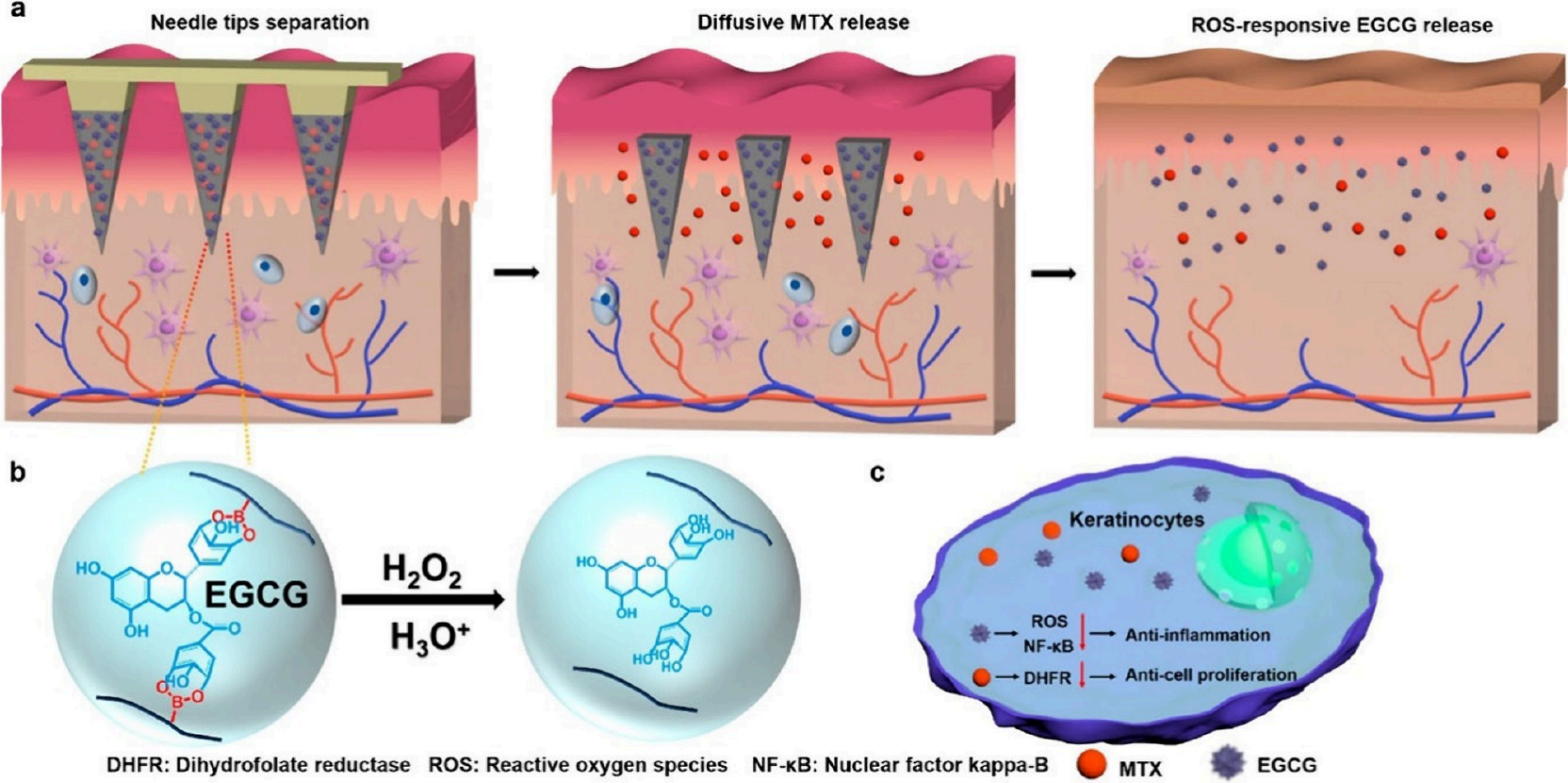
Diagram outlining the operational principle of MN patches exhibiting dual-mode drug release kinetics and the ability to scavenge ROS with the aim to extend and improve the treatment of psoriasis. Reproduced with permission from (156). Copyright 2023 American Chemical Society.

Conventional MNs often exhibit limited drug retention because of the absence of interactions between drugs and polymer materials. Conversely, MNs constructed from stimuli-responsive materials can adjust drug release rates on the basis of the severity of the disease, potentially increasing therapeutic efficacy. In this context, Bi and colleagues developed H_2_O_2_-responsive hydrogel MNs loaded with EGCG and methotrexate for the intelligent delivery of therapeutics in response to ROS in the psoriatic lesion bed for effective management (Figure 9).^156^ In this study, the authors employed a cross-linking technique in which a 10:1 ratio of polyvinylpyrrolidone (PVP) to PVA was used to fabricate responsive hydrogel MNs. The resulting MNs exhibited pyramid-shaped structures measuring 10 × 10 with heights and base widths of 650 and 200 μm, respectively. The cross-linked MN patches were loaded with EGCG and methotrexate (MTX) at concentrations of 157 μg and 5.6 μg, respectively. Furthermore, these MNs demonstrated dual-mode drug release, swiftly delivering MTX through diffusion (98% at 2 h) and sustainably releasing EGCG in response to H_2_O_2_ (68% at 48 h in PBS and 90% at 48 h in 0.1 mM H_2_O_2_ solution). Rapid MTX release inhibited keratinocyte hyperproliferation, whereas prolonged EGCG release suppressed ROS and NF-κB activation in keratinocytes, resulting in anti-inflammatory effects. Nevertheless, the developed MNs significantly improved therapeutic outcomes in a psoriasis-like mouse model, providing hope for effective psoriasis therapy for prolonged periods.

### 5.5 Hyperpigmentation

Skin hyperpigmentation is a prevalent dermatological issue characterized by an overall worsening of skin tone. This alteration in pigmentation arises from a myriad of internal and external influences, such as hormonal fluctuations, exposure to ultraviolet radiation, acne, eczema, injuries, inflammation, and specific medications.^157^ The management of hyperpigmentation involves a multifaceted approach in which various cellular components are targeted.^158^ Topical medications, with commonly utilized agents derived from natural sources such as herbs and microbes, play a pivotal role in hyperpigmentation management. The notable constituents are hydroquinone, RSV, arbutin, glycolic acid, kojic acid, etc.^159−162^ However, the topical administration of these agents in conventional dosage forms, such as gel, cream, or lotion, results in low skin permeability, which is attributed primarily to the presence of the outermost layer of the skin, the stratum corneum. In this context, studies have been conducted on the delivery of these agents through MN-based dermal delivery systems. The MNs penetrate the stratum corneum, facilitating the delivery of hypopigmentation agents to the target site.^163^

RSV, a naturally occurring polyphenol found in diverse fruits and vegetables, has hypopigmentation properties. RSV inhibits melanin synthesis via direct tyrosinase inhibition and indirect pathways, impacting transcription and posttranscriptional regulation. Its antioxidant attributes extend to keratinocytes, shielding against oxidative damage, delivering anti-inflammatory benefits, and contributing to the regulation of melanocyte function, suggesting a promising strategy for hyperpigmentation treatment.^164,165^ However, the application of RSV is limited by its limited bioavailability, inadequate solubility, and poor skin permeability. Thus, Aung and colleagues developed RSV-loaded PVP and eudragit-based dissolving MNs for enhanced transdermal delivery.^166^ With a height of 600 μm and a base width of 300 μm, the developed 11 × 11 MNs demonstrated a maximum accumulation of RSV in the skin at 1107.42 μg/cm^3^. This has the potential to increase the efficacy of treatment for hyperpigmentation conditions. Although phytochemicals could be a greater alternative for synthetic therapeutic agents, their efficacy is still far behind that of synthetic drugs. Therefore, combining multiple phytochemicals to produce synergistic effects would improve the efficacy against hyperpigmentation. In this context, another study by the same team developed alpha-arbutin and RSV-loaded PVP and eudragit-based dissolving MNs to improve skin depigmentation.^167^ The optimized MN patch, featuring cone-shaped 11 × 11 MNs with a height of 575 μm and a base width of 289 μm, contained approximately 13.4 μg of RSV and 67 μg of alpha-arbutin (both needles and base plate). However, the needles alone contained approximately 70 μg of RSV and 350 μg of alpha-arbutin. The optimized MN patch demonstrated enhanced delivery, yielding 1263 μg/cm^2^ alpha-arbutin and 45 μg/cm^2^ RSV through porcine skin, surpassing the cream formulation. In human subjects, compared with the cream formulation, the optimized MN patch delivered alpha-arbutin 2.6 times more effectively and RSV 1.9 times more effectively over a 24-h period. These results suggest that these MNs could be potential candidates for skin whitening.

Vitamin C, alternatively known as ascorbic acid (AA), is a water-soluble vitamin that is abundant in citrus fruits and vegetables. Scientific investigations have demonstrated its capacity to engage with copper ions, specifically at the active sites of tyrosinase, a crucial enzyme facilitating the conversion of tyrosine into melanin. Through this interaction, vitamin C rigorously inhibits the enzymatic action of tyrosinase, thereby intricately inhibiting the process of melanin formation.^168^ However, it suffers from poor skin permeability. To overcome this issue, Zhang and co-workers developed AA- and vitamin A-loaded HA-based MNs for treating skin hyperpigmentation.^169^ With a grid of dimensions of 10 × 10 and a height of 600 μm, the fabricated MNs carried a payload of 90 μg of AA and 27.4 μg of vitamin A. Within 4 h, the release of AA reached 80%, while the release of vitamin A reached 19%. Both substances subsequently achieved complete release by 9 h. Moreover, in white guinea pigs, after four weeks of MN application, the melanin index in the skin epidermis was only 35.23% of the original value, as opposed to that in the control group (solution form). The noticeable disparity in color before and after MN treatment underscores the superior therapeutic efficacy of these dual drug-loaded MNs.

### 5.6 Alopecia

Alopecia, characterized by hair loss, is the second most widespread social ailment and significantly impacts psychological well-being and overall quality of life.^170^ Over the past few years, the incidence of alopecia has noticeably increased; alopecia affects approximately 70% of men, 40% of women and up to 2% of the global population.^171^ Minoxidil and finasteride are the primary medications recommended for treating alopecia. Minoxidil, the predominant topical agent in alopecia treatment, requires prolonged usage for observable efficacy. However, its effectiveness is limited due to the low rate of transdermal absorption.^172^ Notably, finasteride, a type II 5α-reductase inhibitor, is administered orally. However, prolonged use of finasteride may lead to androgen-dependent complications, including side effects such as depression, diminished libido, and disorders in ejaculation.^173^ In recent years, herbs have been used to treat alopecia. However, contemporary trends are shifting toward a more modern approach, focusing on the extraction of bioactive compounds from diverse herb species.^174^ This progressive strategy seeks to leverage functional chemicals obtained from plants as active ingredients for combating alopecia. The utilization of phytochemicals holds promising potential for improving long-term compatibility with the skin in the treatment of alopecia.^175−177^ Although phytochemicals can combat alopecia without many side effects compared with synthetic drugs, their delivery to hair follicles still faces challenges due to their physicochemical properties. Thus, MN-based drug delivery to hair follicles has garnered increasing attention. MNs can directly deliver phytochemicals to hair follicles, increasing their localization. Moreover, MNs enhance the regrowth of hair by activating stem cells in the dermal papilla/hair follicle, increasing the levels of cytokines related to keratin, and increasing the levels of proteins involved in the Wnt/β-catenin pathway.^178,179^

**Figure 10:**
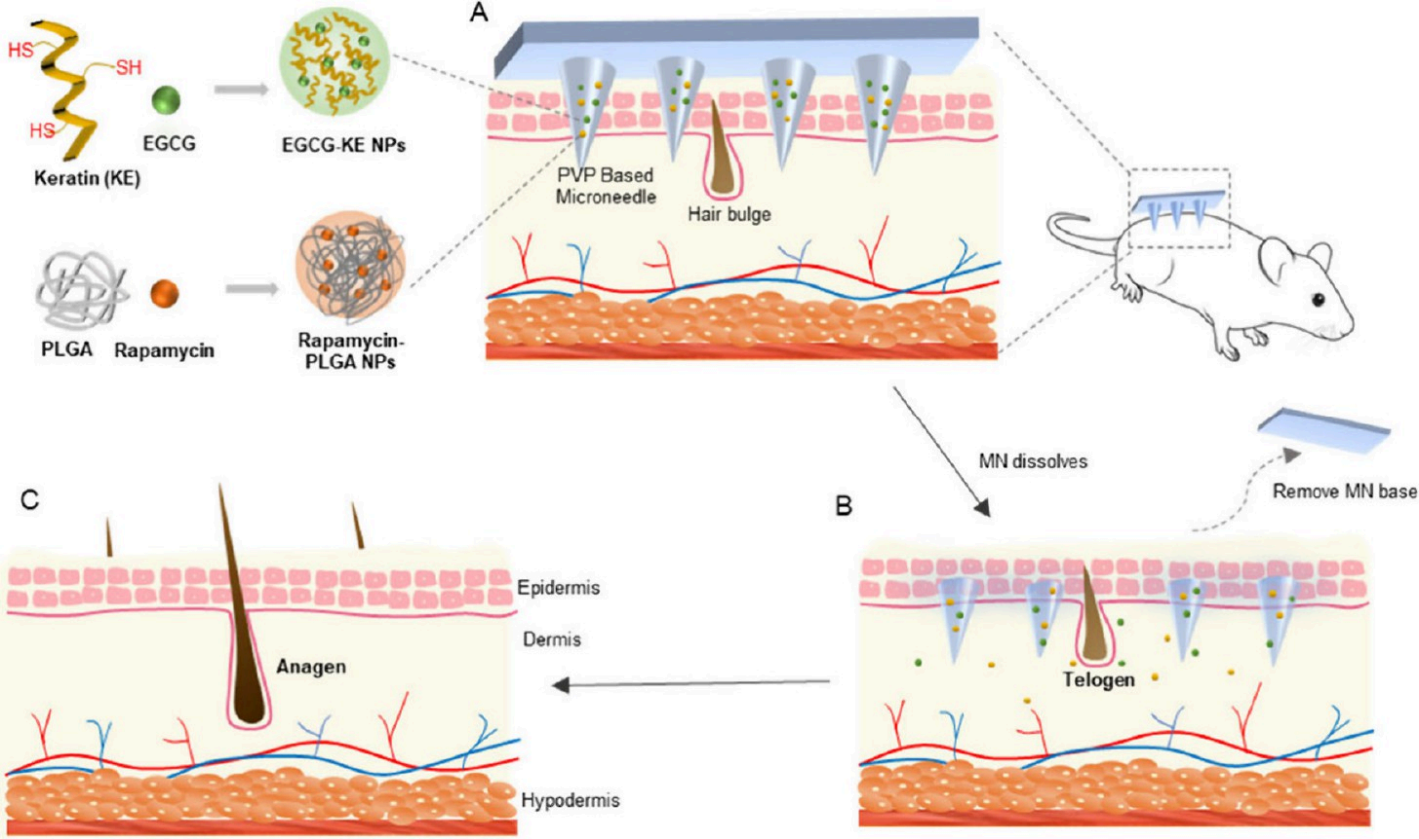
Diagram illustrating the MN device designed for promoting hair regrowth. (A) The DMN, composed of PVP-based MNs loaded with RAPA-PLGA and EGCG-KE NPs, penetrates the skin. (B) The MNs dissolve quickly and release nanoparticles to facilitate drug transport into the hair follicle niche. (C) Drugs can activate the healthy hair cycle, hasten the transition of hair follicles from the telogen phase to the anagen phase, and stimulate hair regrowth. Reproduced from ref (183), licensed under CC BY 4.0. Copyright 2022 The Authors.

EGCG, the primary catechin found in green tea, has been shown to promote hair growth through various mechanisms. These include the regulation of phosphorylated Erk and Akt, an increase in the ratio of Bcl-2/Bax,^180^ the modulation of the sonic hedgehog and protein kinase B signaling pathways,^181^ and the selective inhibition of 5α-reductase activity.^182^ However, they suffer from limited skin permeability. Additionally, the use of multiple therapeutic agents significantly improved hair growth compared with the use of individual agents. In this context, Lin and teammates developed EGCG- and rapamycin NP-loaded dissolvable MNs for the promotion of hair growth (Figure 10).^183^ With a 10 × 10 array, cone-shaped MNs with a height of 680 μm and a base width of 320 μm contained 0.1 μg of rapamycin and 4 μg of EGCG. Moreover, the developed MN patch exhibited superior therapeutic efficacy compared with topical application and individual delivery of rapamycin and EGCG via MNs during a 15-day treatment period in C57BL/6J mice.

**Figure 11:**
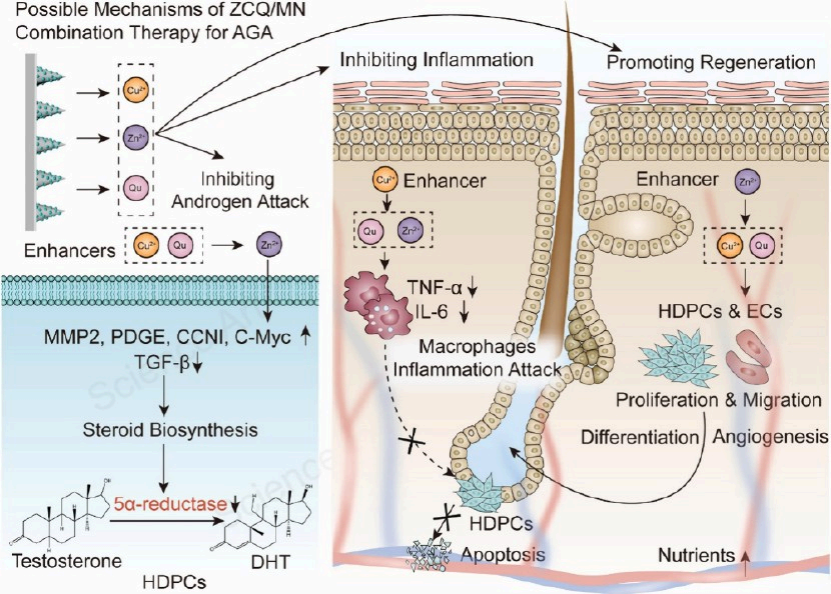
Potential mechanism behind the combined ZCQ/MN therapy for androgenetic alopecia (AGA): (a) zinc ions (Zn^2+^) hinder the detrimental effects of dihydrotestosterone (DHT) on hair follicle stem cells (HDPCs), whereas quercetin (Qu) and copper ions (Cu^2+^) increase the effectiveness of Zn^2+^. (b) Zn^2+^ and Qu synergistically deter inflammation and safeguard hair follicle stem cells against inflammation-induced death with Cu^2+^ enhancing the effects of Zn^2+^ and Qu. (c) Cu^2+^ and Qu in combination both increase the proliferation and migration of hair follicle stem cells and activate endothelial cells (ECs), thereby inducing angiogenesis to increase nutrient supply and fostering hair growth with Zn^2+^ amplifying the effects of Cu^2+^ and Qu. Reproduced from ref (186), licensed under CC BY 4.0. Copyright 2022 The Authors.

Quercetin, a flavonoid phytochemical, is commonly found in a variety of fruits and vegetables. Research indicates that quercetin stimulates the growth of resting hair follicles by promoting rapid proliferation of follicular keratinocytes.^184^ More specifically, the activation of the MAPK/CREB signaling pathway promotes hair growth.^185^ Furthermore, essential trace elements such as zinc (Zn^2+^) and copper (Cu^2+^) are crucial for normal hair follicle development and the regulation of immune cells. Therefore, Zhang and colleagues developed a quercetin-encapsulated, Zn^2+-^ and Cu^2+^-doped, mesoporous silica nanoparticle-loaded dissolving MN patch for combinatorial therapy for alopecia.^186^ The fabricated 20 × 20 array of MNs, each with a height of 600 μm, demonstrated the ability to effectively administer loaded therapeutics to a depth of 300 μm in mice, resulting in a noteworthy increase in hair growth. The mechanism underlying the promotion of hair follicle regeneration primarily includes the mitigation of inflammatory responses, facilitation of angiogenesis, inhibition of dihydrotestosterone, and activation of hair follicle stem cells (Figure 11). Furthermore, this study illustrates that a methodical approach focusing on various pathophysiological aspects of androgenetic alopecia utilizing a blend of phytochemical (quercetin) and bioactive metal ions (Zn^2+^ and Cu^2+^) constitutes an efficacious therapeutic strategy for mitigating hair loss.

### 5.7 Scar

Scars manifest as atypical alterations in skin appearance, originating from the irregular arrangement of collagen due to physical injuries such as burns, surgical procedures, and acne. These scars, which form because of injury, replace lost cells on the skin.^187^ The rapid formation of fibrotic tissue during the healing process determines whether the scar takes the form of a keloid or hypertrophic tissue. Existing clinical interventions for scars, including surgery, laser therapy, cryotherapy, pressure therapy, radiotherapy, and topical therapy (immunomodulatory agents, corticosteroids, etc.), often prove expensive, painful, and ineffective and may yield significant adverse effects over time.^188^ Therefore, numerous studies have identified the potential of plant-derived compounds as viable treatments for preventing scarring. For example, CUR has been demonstrated to mitigate scar formation by inhibiting fibroblast proliferation.^189^ However, the primary challenge lies in delivering these phytochemicals efficiently to the dermal layer, a task complicated by conventional formulations such as gels and creams. Therefore, MN-based systems have been considered for the efficient delivery of phytochemicals to target sites, ensuring effective scar management. Additionally, MN treatment enhances type I collagen, glycosaminoglycans, and pivotal growth factors (VEGF, FGF-7, EGF, and TGF-β), promoting collagen synthesis and neovascularization. At the histological level, MN induces epidermal thickening, elevated dermal collagen, and increased elastic fiber deposition, culminating in tightened skin and diminished scarring over a span of weeks to months.^190−193^

**Figure 12:**
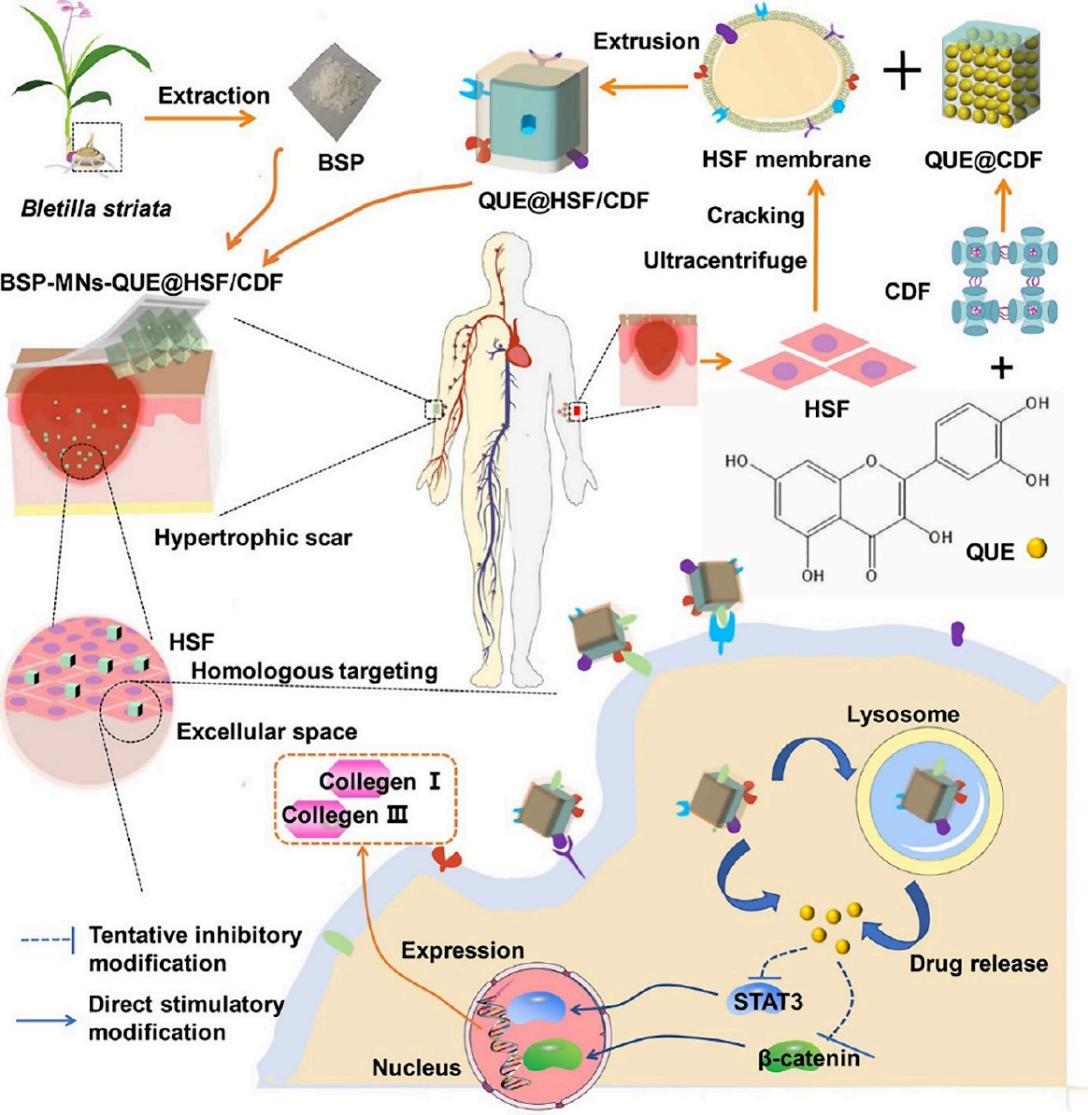
Schematic illustration of the fabrication and administration of quercetin-encapsulated cyclodextrin metal organic framework (C-MOF)-loaded *Bletilla striata* polysaccharide-based dissolving MNs for hypertrophic scar management. Reproduced with permission from ref (197). Copyright 2021 American Chemical Society.

Quercetin has demonstrated promising effects in vitro, specifically in reducing the proliferation of fibroblasts derived from hypertrophic scars.^194,195^ Additionally, it has been found to modulate intracellular signaling pathways and influence collagen synthesis. Studies have shown that quercetin not only arrests the cell cycle, thereby inhibiting fibroblast proliferation but also hinders the contraction of the fibroblast-populated collagen lattice (FPCL).^196^ A recent study conducted by Wu and colleagues reported quercetin-encapsulated cyclodextrin metal organic framework (C-MOF)-loaded *Bletilla striata* polysaccharide-based dissolving MNs (Q@C-MOF@MNs) for the management of hypertrophic scars (Figure 12).^197^ The incorporation of quercetin into the C-MOF increased its solubility from 10 μg/ml to 342 μg/ml, representing a 30-fold increase. The MN array is arranged in a 10 × 10 pattern, with each needle shaped like a quadrangular tower. The base of each needle measures 230 μm on each side, and it stands at a height of 380 μm (with the tip extending to 230 μm). The developed Q@C-MOF@MN formulation enhances therapeutic effects on hypertrophic scar-bearing rats by enhancing MN permeation, enhancing hypertrophic scar fibroblast targeting, enhancing quercetin solubilization by C-MOF, enhancing synergistic *Bletilla striata* polysaccharide efficacy, modulating the Wnt/β-catenin and JAK2/STAT3 pathways, and reducing collagen I and III expression in hypertrophic scars. Overall, this could be a promising strategy for the effective treatment of scars. However, further clinical studies are warranted.

Shikonin, which is extracted from *Arnebiae Radix* (TCM), effectively inhibits cell viability, proliferation, and collagen production in fibroblasts derived from scars and is thus a promising alternative for scar treatment. However, its inadequate skin permeability hinders its therapeutic efficacy. Thus, Ning and Teammates developed shikonin-loaded HA-based MNs for treating hypertrophic scars.^198^ The developed 10 × 10 array MNs, with a height of 1000 μm and a base width of 300 μm, were loaded with 30.76 μg of shikonin. Moreover, shikonin@HA-MNs can regulate the viability and metabolic activity of TGFb1-treated hypertrophic scar fibroblasts, revealing a localized therapeutic impact that holds promise for site-specific scar treatment. Additionally, the application of shikonin@HA-MNs has been shown to suppress the expression of scar-related genes such as TGFb1, FAP-a, and COL1A1. Collectively, these findings underscore the significant potential of shikonin@HA-MNs for effective scar management.

### 5.8 Atopic Dermatitis

Atopic dermatitis (AD) presents as an allergic skin condition marked by intricate symptoms, including skin dryness and thickening, accompanied by frequent scratching.^199,200^ In 2022, the International Eczema Council reported that approximately 223 million individuals suffer from AD, as per the Global Burden of Disease (GBD) 2022. Among these individuals, approximately 43 million are aged 1–4 years.^201^ Addressing AD involves a multifaceted treatment approach involving various pharmacological therapies. The key components of this treatment arsenal include topical calcineurin inhibitors, antihistamines, antibiotics, topical corticosteroids, systemic immunosuppression, etc. However, the effectiveness of these interventions is intricately tied to the potential occurrence of side effects.^202^ Thus, phytochemicals offer promising prospects for the effective treatment of AD, as supported by several scientific reports emphasizing their safety and efficacy.^203^ Furthermore, recent studies have indicated that the delivery of phytochemicals via MN-based systems significantly enhances outcomes in the treatment of AD.^204^

**Figure 13:**
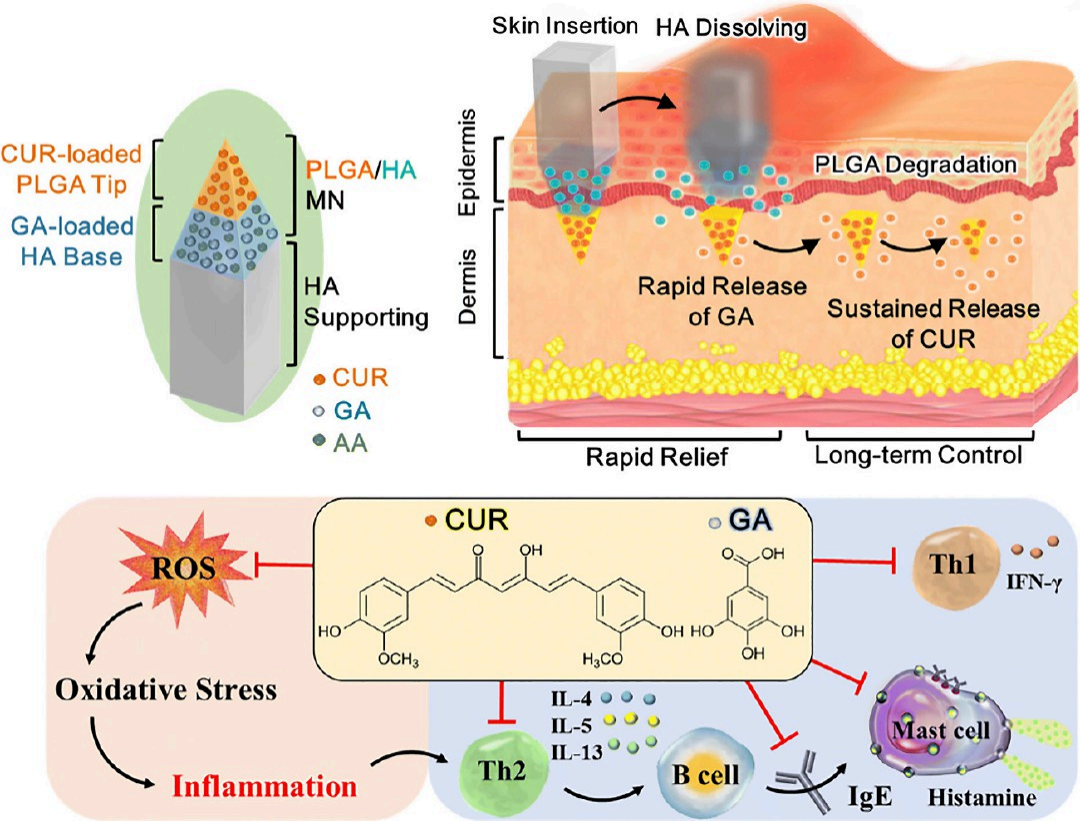
Visual representation of a double-layered patch made of poly(lactic-co-glycolic acid) (PLGA) and sodium hyaluronate (HA), which serves as a sustained-release system for polyphenols to effectively manage atopic dermatitis (AD) both rapidly and over the long-term. When inserted into the skin, the HA layer dissolves within 5 min, facilitating the release of gallic acid (GA), while the PLGA layer remains embedded in the dermis, gradually releasing curcumin (CUR) over a period of 2 months. The simultaneous delivery of CUR and GA has synergistic antioxidant and anti-inflammatory effects, which effectively reduces the oxidative stress-induced production of proinflammatory cytokines and alleviates symptoms associated with AD. Reproduced with permission from ref (205). Copyright 2023 Royal Society of Chemistry.

Polyphenols, which are prevalent in fruits, vegetables, and herbs, possess strong anti-inflammatory and antioxidant properties and have potential in the prevention and treatment of various diseases, particularly chronic inflammation. They function by rebalancing redox levels to combat oxidative stress and regulating inflammatory reactions through the inhibition of proinflammatory pathways. Therefore, polyphenolic compounds such as CUR and gallic acid (GA) could be potential candidates for treating AD. However, these compounds suffer from poor skin permeability. To overcome this issue, Chen and colleagues developed dual-layered MNs based on PLGA and HA loaded with CUR and GA for prolonged treatment of AD (Figure 13).^205^ The developed array of 9 × 9 pyramidal MNs, with a height of 600 μm and base width of 300 μm, exhibited loading of 225 μg of CUR and 117 μg of GA. Upon penetrating the skin, the HA layer dissolves quickly within 5 min, stimulating the release of GA. Moreover, the PLGA tip remained lodged in the dermis, ensuring the continuous release of CUR over a period of 2 months. At the outset, both GA and CUR are released concurrently from the MNs, initiating a concerted action to deliver synergistic anti-inflammatory and antioxidant effects, promptly alleviating symptoms of AD. Following the full release of GA, the sustained release of CUR ensures the preservation of the achieved effect for a minimum of 56 days. Interestingly, compared with CUR@MNs, GA/CUR@MNs not only swiftly diminished the dermatitis score starting on day 2 but also markedly curbed epidermal hyperplasia and the buildup of mast cells. These results highlight the efficacy of the dual-phytochemical-loaded dual-layered MN patch as a promising approach for the effective management of AD over both short and extended durations.

Research indicates that EGCG holds promise as a therapeutic remedy for AD owing to its anti-inflammatory and antioxidative properties.^206^ The bioavailability of oral EGCG is notably low, ranging from approximately 0.1% to 0.3%, which is attributed primarily to inadequate absorption and swift metabolism in the gastrointestinal tract. Furthermore, its vulnerability to degradation when exposed to heat and light imposes limitations on both its shelf life and therapeutic outcomes. Thus, Chiu and teammates developed polyglutamate-based MNs loaded with EGCG and AA for the management of AD.^207^ The loaded AA helps to stabilize the unstable EGCG. The developed MN patch contained a total of 81 pyramid-shaped needles with heights and base widths of 600 and 300 μm, respectively. Furthermore, the MN patches contained 438 μg and 358 μg of AA and EGCG, respectively. Over a span of 4 weeks, administering EGCG/AA@MNs once a week to a mouse model of AD led to significant improvement in skin lesions and a reduction in epidermal hyperplasia. This was evidenced by notable decreases in serum IgE levels and histamine levels, along with the inhibition of IFN-γ and Th2-type cytokine production, compared with those in the untreated AD group. Remarkably, the efficacy of once-weekly MN therapy was comparable to that of daily topical application of an EGCG and AA solution, yet with a significantly reduced frequency of administration and required dosage, making these MNs excellent choices for AD treatment.

### 5.9 Other Conditions

**Figure 14:**
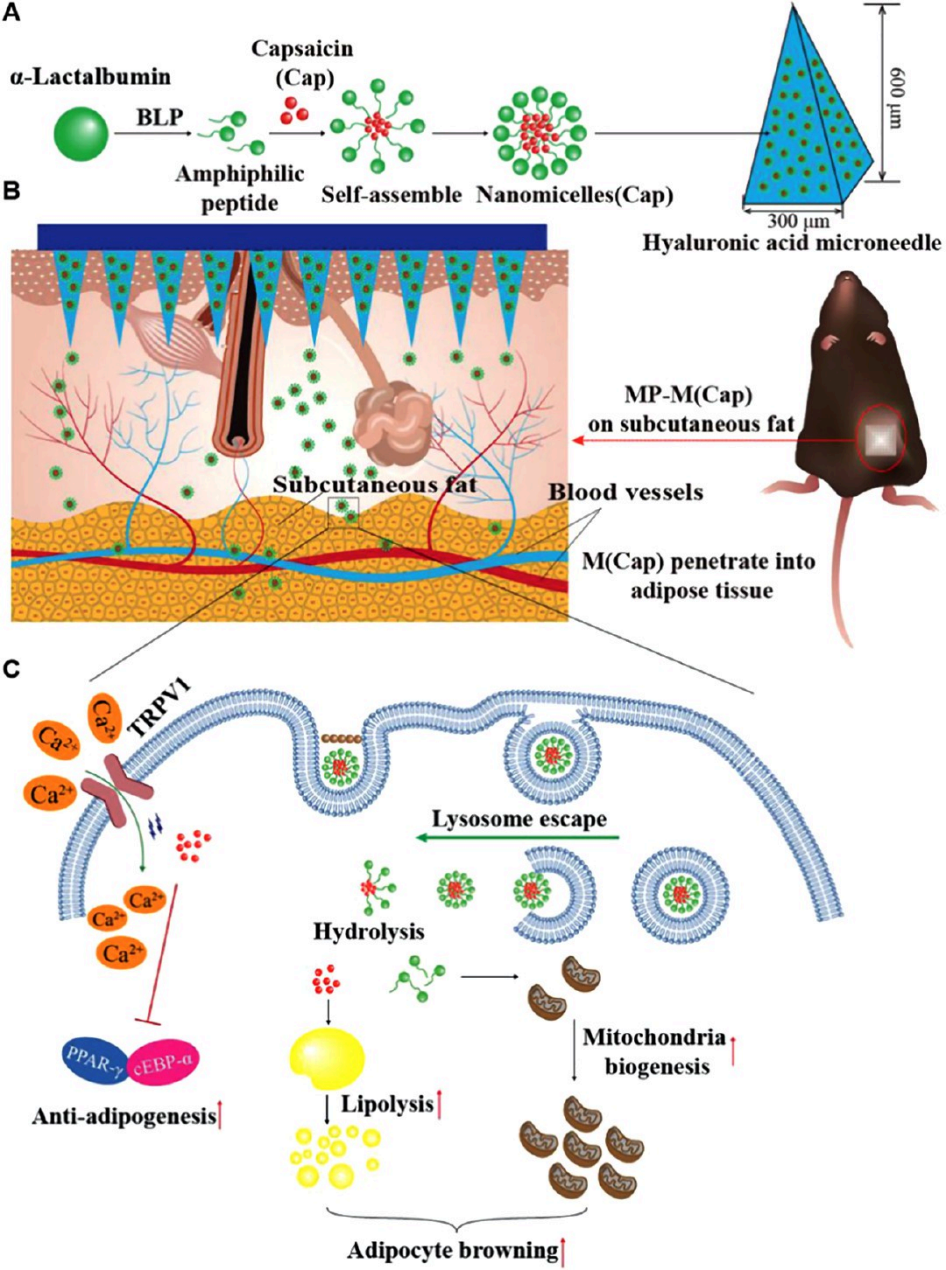
Diagram depicting a versatile MN patch incorporating capsaicin-loaded micelles designed to inhibit adipogenesis and induce adipocyte browning. (A) Micelles containing capsaicin [M(Cap)] were formulated through the self-assembly of α-lactalbumin peptides and capsaicin and then incorporated into an MN patch composed of hyaluronic acid (HA) and polyvinyl alcohol (PVA) with the ability to melt at body temperature. (B) The MN patch loaded with M(Cap) was applied to the skin devoid of fur on the left abdominal subcutaneous fat of obese mice induced by a high-fat diet (HFD). (C) M(Cap) penetrates adipose tissue where it is internalized and modulates adipocyte lipogenesis while promoting the browning of white adipocytes. Reproduced with permission from ref (212). Copyright 2021 Wiley.

Obesity, which is characterized by excessive buildup of white adipose tissue (WAT), is linked to various harmful conditions, including oxidative stress, inflammation, hypoxia, and disruptions in adipokine secretion within the adipose tissue. These factors collectively contribute to systemic inflammation and metabolic disturbances, leading to conditions such as hyperlipidaemia and fatty liver disease. Consequently, individuals with obesity are at heightened risk of developing severe health complications, including cardiovascular diseases, cancer, and diabetes.^208−210^ Recently, researchers have developed MN patches aimed at combating obesity by delivering therapeutic agents painlessly and directly into subcutaneous WAT (sWAT). This approach induces a process called browning, where white adipocytes are transformed into energy-burning brown-like adipocytes.^211^ For example, Bao and colleagues encapsulated capsaicin, a naturally occurring alkaloid derived from chili peppers of the genus Capsicum, into nanomicelles and further loaded them into an HA-based MN patch for delivery to adipocytes, aiming to manage obesity (Figure 14).^212^ However, these traditional MNs, which are typically made of polymer matrices, have a limited capacity to encapsulate therapeutics.

**Figure 15:**
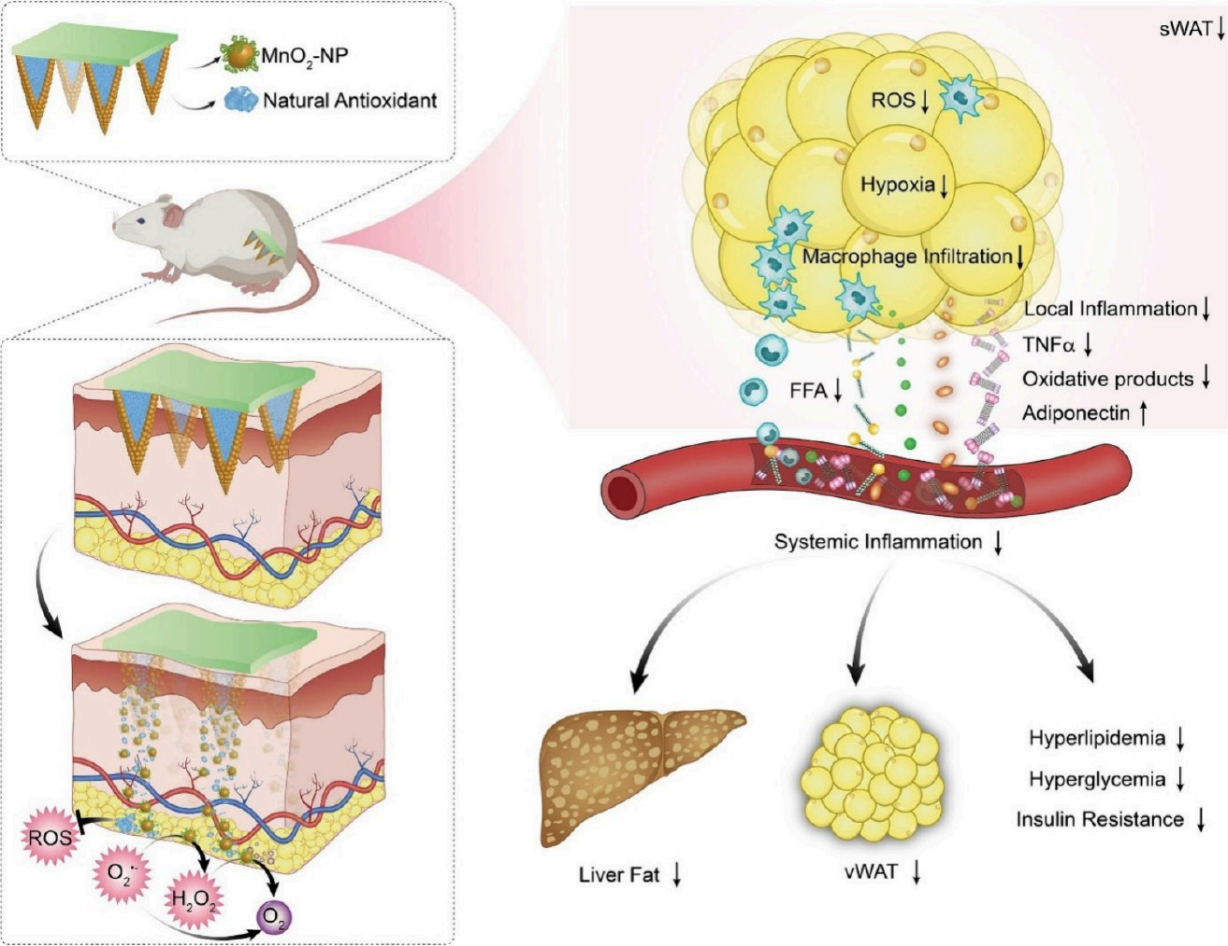
Visual depiction of transdermal anti-inflammatory treatment using dry powder microneedles (MNs) aimed at restructuring subcutaneous fat by alleviating oxidative stress, hypoxia, and inflammation, thereby enhancing systemic metabolism. Reproduced with permission from ref (213). Copyright 2024 Elsevier.

To overcome the drawbacks of previous studies, Zan and colleagues developed novel core–shell MNs composed entirely of compact dry powders of therapeutic agents (Figure 15).^213^ Here, the authors crafted the outer layer of MNs from manganese dioxide nanoparticles (MnO_2_ NPs), and the inner core was loaded with RSV, a natural antioxidant agent. MnO_2_ NPs possess the remarkable ability to scavenge harmful ROS such as hydrogen peroxide (H_2_O_2_) and superoxide anion (O2^•–^). Furthermore, RSV synergizes with this effect. The developed novel dry powder MNs facilitate transdermal anti-inflammatory therapy, effectively remodelling targeted obese sWAT. This intervention not only leads to a reduction in WAT mass but also yields substantial improvements in overall metabolic health. These benefits are demonstrated through the complete resolution of type 2 diabetes symptoms, the mitigation of systemic inflammation and hyperlipidemia, and a significant reduction in ectopic fat accumulation in organs such as visceral WAT (vWAT) and the liver, making the developed MN patch a promising platform for the effective management of obesity. Table 1 shows recent research findings on MN-mediated phytochemical delivery for managing various conditions.

**Table 1:** Investigations on MN-Mediated Delivery of Phytochemicals for Managing Various Conditions

applications	type of MNs	dimensions	phytochemical/other therapeutic agent	amount of phytochemical/MN patch^a^	ref
Triple-negative breast cancer	Dissolving MNs	12 × 12 array, 1200 μm height, and 300 μm base width	Paclitaxel/anti-PD-1	150 μg	(110)
Melanoma	Dissolving MNs	12 × 12 array, 800 μm height, and 300 μm base width	Paclitaxel	54 μg	(214)
Melanoma	Hydrogel MNs	11 × 11 array, 600 μm height, and 300 μm base width	Curcumin	NM	(107)
Wound healing	Hydrogel MNs	15 × 15 array, 345 μm height and 250 μm base width	Curcumin	122 μg	(133)
Wound healing	Hydrogel MNs	20 × 20 array, 770 μm height, and 410 μm base width	Tannic acid	NM	(127)
Wound healing	Hydrogel/Dissolving MNs	15 × 15 array, 500 μm height, and 350 μm base width	Asiatic acid	NM	(215)
Polymicrobial biofilms-infected wounds	Hollow MNs (AdminPen 1500)	1400 μm height	carvacrol		(32)
Rheumatoid arthritis	Dissolving MNs	15 × 15 array, 500 μm height	Tetrandrine	NM	(141)
Gout	Dissolving MNs	20 × 20 array, 525–542 μm height and 353–370 μm base width	Colchicine	48 μg	(216)
Gout	Dissolving MNs	12 × 12 array, 800 μm height and 300 μm base width	Colchicine	30–35 μg	(144)
Gout	Dissolving MNs	16 × 16 array, 850 μm height and 300 μm base width	Colchicine	2.8 mg	(31)
Psoriasis	Dissolving MNs	600 μm height and 300 μm base width	Epigallocatechin-3-gallate/Dexamethasone	NM	(155)
Psoriasis	Hydrogel MNs	10 × 10 array, 650 μm height and 200 μm base width	Epigallocatechin-3-gallate/Methotrexate	157 μg/5.6 μg	(156)
Hyperpigmentation	Dissolving MNs	11 × 11 array, 600 μm height and 300 base width	Resveratrol	NM	(166)
Hyperpigmentation	Dissolving MNs	11 × 11 array, 575 μm height and 289 μm base width	Resveratrol/alpha-arbutin	13.4 μg/67 μg	(167)
Hyperpigmentation	Dissolving MNs	10 × 10 array, 600 μm height and 300 μm base width	Ascorbic acid/Vitamin A	90 μg/27.4 μg	(168)
Alopecia	Dissolving MNs	10 × 10 array, 680 μm height and 320 μm base width	Epigallocatechin-3-gallate/Rapamycin	4 μg/0.1 μg	(183)
Alopecia	Dissolving MNs	20 × 20 array, 600 μm height	Quercetin	NM	(186)
Hypertrophic scar	Dissolving MNs	10 × 10 array, 610 μm height and 230 μm base width	Quercetin	NM	(197)
Hypertrophic scar	Dissolving MNs	10 × 10 array, 1000 μm height and 300 μm base width	Shikonin	30.76 μg	(198)
Atopic dermatitis	Dissolving MNs	9 × 9 array, 600 μm height and 300 μm base width	Curcumin/Gallic acid	225 μg/117 μg	(205)
Atopic dermatitis	Dissolving MNs	9 × 9 array, 600 μm height and 300 μm base width	Epigallocatechin-3-gallate/Ascorbic acid	358 μg/438 μg	(207)
Obesity	Dissolving MNs	10 × 10 array, 600 μm height and 300 μm base width	Capsaicin	NM	(212)
Obesity	Dissolving MNs	10 × 10 array, 1000 μm height and 300 μm base width	Resveratrol	8 μg	(213)
Obesity	Dissolving MNs	10 × 10 array, 600 μm height and 300 μm base width	Rutin	8 mg	(217)
Malaria	Dissolving MNs	11 × 11 array, 800 μm height	Artemether/Lumefantrine	NM	(218)
Malaria	Dissolving MNs		Artemether/Lumefantrine	179 μg/106 μg	(219)
Malaria	Dissolving MNs	19 × 19 array, 600 μm height and 300 μm base width/14 × 14 array, 600 μm height and 400 μm base width	Artemether/Lumefantrine	1580 μg/1500 μg	(33)
Malaria	Dissolving MNs	13 × 13 array, 680 μm height and 380 μm base width	Artemether	600 μg	(220)
Acne vulgaris	Dissolving MNs	12 × 12 array, 500 μm height and 300 μm base width	Azelaic acid/Matrine	201 μg/259 μg	(221)
Anti-inflammatory and analgesic effects	Solid MNs	250 μm height and 100 μm base width	Total alkaloids isolated from *Aconitum sinomontanum*		(222)
Dysmenorrhea	Dissolving MNs	11 × 11 array, 700 μm height and 360 μm base width	Cinnamon oil	NM	(223)
Intradermal delivery	Dissolving MNs	11 × 11 array, 900 μm height and 300 μm base width	Curcumin	10.9 μg	(30)

a NM: not mentioned in μg or mg.

## 6 MN-Mediated Phytophotosensitizer Delivery for Photodynamic Therapy

PDT represents a contemporary, minimally invasive therapeutic modality utilized for treating a wide array of diseases, encompassing both nononcological conditions and diverse cancer types across different anatomical sites. PDT has demonstrated notable success in oncology, rheumatology, dermatology, etc.^224−226^ In preclinical investigations, local PDT for treating melanoma and nonmelanoma skin cancer has risen to prominence owing to its clear advantages over systemic PDT.^227^ These include reduced targeted tumor eradication with minimal impact on healthy tissues, cumulative systemic toxicity, potential for standalone or supplementary anticancer treatment, and direct administration of PS to the treatment area. This localized strategy eliminates the necessity of avoiding light exposure typically associated with systemic PS administration, thus fostering improved patient adherence and improving quality of life.^228^

**Figure 16:**
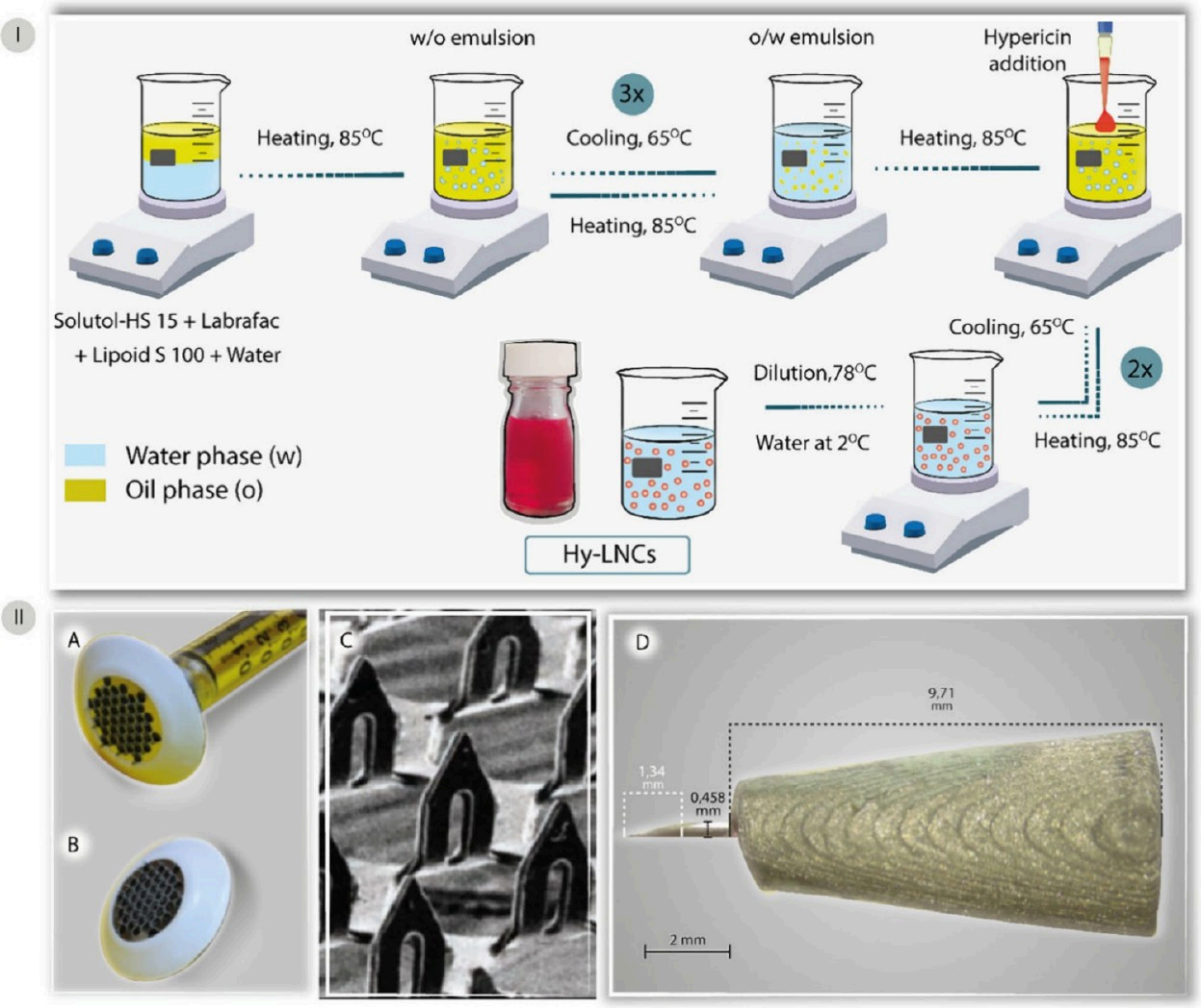
(I) Diagram illustrating the fabrication process of Hy-LNCs via the adapted phase inversion technique. (II) AdminPen apparatus: (A,B) 43 metallic hollow stainless-steel microneedles measuring 1200 μm in length and arranged in a circular microneedle array spanning 1 cm^2^; (C) SEM image (reproduced with authorization from AdminMed); (D) configuration of an individual hollow microneedle featuring a beveled shaft measuring 1300 μm. Reproduced from ref (34), licensed under CC BY 4.0. Copyright 2022 The Authors.

There is a growing focus on plant-derived PSs because of their reduced toxicity in comparison with that of their synthetic counterparts, and they have attracted considerable attention. A recent study conducted by our group engineered hypericin lipid nanocapsules and delivered them intradermally via in-house-developed single hollow MNs (1300 μm) and marketed AdminPen (1200 μm) bearing 43 hollow MNs for the treatment of nonmelanoma skin cancer (Figure 16).^34^ Hypericin, a phenanthroperylenequinone derivative, is among the most powerful photosensitizing agents. It is naturally sourced from *Hypericum perforatum*, commonly known as St. John’s wort. The results from the in vivo assessment using a nude mouse model with transplanted tumors revealed that hypericin lipid nanocapsules delivered via hollow MNs displayed remarkable antitumor efficacy (≈85%) following irradiation with 595 nm light. These findings suggest that the hollow MN-driven delivery of hypericin lipid nanocapsules, followed by irradiation, could represent a minimally invasive and localized approach for managing nonmelanoma skin cancers.

Recently, PDT has emerged as a highly promising treatment for autoimmune diseases such as RA, offering a convenient and noninvasive approach.^229,230^ The goal of PDT is to prompt cell death in inflamed and hyperplastic cells within the joint, effectively curbing the abnormal growth of synovial tissue.^231^ Furthermore, the encapsulation of PS in nanoparticles offers a means to increase their concentration and duration within target tissues, enhancing treatment efficacy. In this context, another study by Abd-El-Azim and colleagues developed hypericin-emulsomes for intra-articular delivery via hollow MNs (AdminPen, 43 needles with 1200 μm) through intradermal administration to treat RA.^232^ The results from the in vivo study involving male Sprague–Dawley rats revealed that the group treated with hypericin-emulsomes via hollow MNs combined with PDT presented no significant differences in terms of joint diameter or the levels of inflammatory cytokines, such as IL-1 and TNF-α, compared with those of healthy rats. These findings emphasize the promising potential of photoactivated hypericin-emulsomes when utilized in conjunction with hollow MNs for managing RA. However, further studies in large animals are warranted before clinical studies can be performed.

## 7 Patents

Numerous scientific journals have highlighted the use of MNs for delivering phytochemicals across various applications. Despite this, only a handful of researchers have pursued patent protection for their innovations, aiming to progress phytochemical-loaded MNs into clinical trials and subsequent commercialization. Table 2 shows a curated collection of recently published patents on the utilization of MNs for the intra/transdermal delivery of phytochemicals for diverse applications.

**Table 2:** Patents Published on MN-Mediated Phytochemical(s) Delivery for Various Applications (Source: Espacenet.com)

patent no.	inventors	title of invention	phytochemical/s	application	year
US2015290163A1	Quan et al.	Retinoic acid microneedle	Retinoic acid	Wrinkles	2015
CN104382884A	Gao et al.	Preparation method of intradermal delivery microneedle preparation of artemisinin derivative	Artemisinin derivative	Malaria	2015
CN108904299A	Li et al.	Soluble micro needle with antiacne effect and preparation method thereof	Epigallocatechin gallate	Acne vulgaris	2018
CN111920699A	Wen et al.	Whitening and freckle-removing composition, whitening and freckle-removing soluble microneedle patch and preparation method thereof	Resveratrol	Skin whitening	2020
CN113546153A	Chen	Antiaging composition capable of activating cells and preparation method thereof	Resveratrol/Ascorbic acid	Antiaging	2021
WO2021143951A2	Ding et al.	Colchicine soluble microneedle patch and preparation method therefor	Colchicine	Gout	2021
CN115054732A	Mao et al.	Suture-free multilayer drug-loaded myocardial patch and preparation method thereof	Curcumin	Myocardial infarction	2022
WO2022142195A1	Li et al.	Microneedle patch and preparation method therefor	Resveratrol	Antiaging	2022
KR20220037994A	Kim et al.	Soluble microneedle for transdermal delivery containing ascorbic acid derivative manufacturing method thereof and transdermal delivery patch comprising the same	Ascorbic acid	Transdermal delivery	2022
CN114259479A	Chen and Hua	Microneedle, microneedle patch and preparation method thereof	Dihydromyricetin/Hesperetin	Antiaging/Skin whitening	2022
CN116509744A	Wang et al.	Microneedle containing curcumin-zinc MOF and application of microneedle in hair growth promotion	Curcumin	Alopecia	2023
US2023404929A1	Ozdoganlar et al.	Intradermal Delivery of Extracellular Vesicle-Encapsulated Curcumin Using Dissolvable Microneedle Arrays	Curcumin	Intradermal delivery	2023
CN116672275A	Gao et al.	Whitening antioxidation composition, whitening antioxidation product prepared from same and preparation method of whitening antioxidation product	Resveratrol/Azelaic acid	Skin whitening	2023
CN115634215A	Wang et al.	Hydrothermal response microneedle patch as well as preparation method and application thereof	Paclitaxel	Superficial tumor (Breast/skin cancer)	2023
CN116687833A	Shuai et al.	Soluble microneedle containing antibody coupled albumin paclitaxel nanodrug as well as preparation method and application of soluble microneedle	Paclitaxel	Superficial tumor (Breast/skin cancer)	2023
US2023145564A1	Khademhosseini et al.	Gelatin methacryloyl-based microneedle patches for delivery of water-insoluble drugs	Curcumin	Melanoma	2023
WO2024051132A1	Peng et al.	Colchicine hydrogel microneedle and preparation method therefor	Colchicine	Gout	2024
CN117338922A	Qu et al.	Sinomenine and total glucosides of paeony loaded photothermal driving motor microneedle for treating rheumatoid arthritis and preparation method thereof	Sinomenine	Rheumatoid arthritis	2024

## 8 Conclusion and Future Perspectives

Phytochemicals are various primary and secondary metabolites found within plants and are known for their intricate chemical structures. Their versatility and potential in modern therapeutic interventions have been demonstrated for diverse disease conditions, such as cancer, psoriasis, arthritis, alopecia, dermatitis, obesity, and chronic wounds. MN-based drug delivery systems provide enhanced intra/transdermal delivery of phytochemicals, surpassing conventional formulations. According to BCC Research LLC’s market analysis, the worldwide market for MNs is projected to increase from $1.0 billion in 2023 to $1.3 billion by 2028, with a CAGR of 5.1% from 2023 to 2028.^233^ MNs ensure precise and consistent delivery of phytochemicals into dermal layers, optimizing therapeutic outcomes via controlled release and increased bioavailability. They offer prolonged therapeutic potential, reduced administration frequency, and improved patient compliance. Compared with oral or parenteral administration, MNs bypass first-pass metabolism, minimizing systemic side effects while enabling rapid onset of action. Moreover, they enhance skin permeability, mitigate the risk of irritation, and allow for combination therapy, fostering synergistic effects in various medical applications. Moreover, the MN formulation offers a potential solution to the stability issues faced by certain phytochemicals, which are prone to hydrolysis. This degradation process can pose significant challenges when these phytochemicals are incorporated into conventional formulations, such as gels, creams, or lotions, especially during long-term storage. MN technology has secured a prominent position on the “10 Emerging Technologies 2020” list with the title “Microneedles for Painless Injections and Tests” curated by the World Economic Forum.^234^

Although MN technology has demonstrated significant promise as a drug delivery platform in preclinical studies, it has faced several challenges in clinical translation. The launch of the first MN-based product, Qtrypta, was halted after the FDA rejected it in 2020 because of inconsistencies in drug plasma levels across manufacturing batches.^235^ This underscores the importance of improving manufacturing processes to ensure product consistency. Similarly, MNs have shown promise in delivering various phytochemicals for treating numerous conditions on the basis of in vitro and preclinical studies in small animal models, but further preclinical studies in large animal models are needed before they enter clinical trials. To achieve this efficiently, collaboration among academia, industry, nonprofits, and hospitals is crucial to bridge the gap between research and patient care. The mission of PATH’s “Microarray Patches-Centre of Excellence” is to expedite the expansion of MN manufacturing and enhance the efficiency of regulatory processes.^236^ Health authorities must establish clear definitions and standards for MN drug delivery. Additionally, further investigational studies are essential to thoroughly evaluate the efficacy and long-term safety profiles of MNs. However, several companies, including Micron Biomedical, CosMED Pharmaceutical, Raphas, Micropoint Technologies, and QuadMedicine, are constantly working on the use of polymeric MNs for various pharmaceutical, vaccine, and cosmetic applications. In summary, these advancements are anticipated to lead to the availability of commercially viable MNs containing phytochemicals, catering to a diverse range of therapeutic applications in the near future.

## Data Availability

The data sets generated during and/or analyzed during the current study are available from the corresponding author on reasonable request.
